# Supplementary material for: Synthetic Study toward Triterpenes from the Schisandraceae Family of Natural Products
Source: Molecules. 2023 May 31;28(11):4468. doi: 10.3390/molecules28114468 (PMC10254518; doi:10.3390/molecules28114468)
Supplement: Supplementary file 1 [file molecules-28-04468-s001.zip › molecules-2401301-supplementary.pdf]

Supporting Information

NMR spectra of novel compounds

¹H 400 MHz

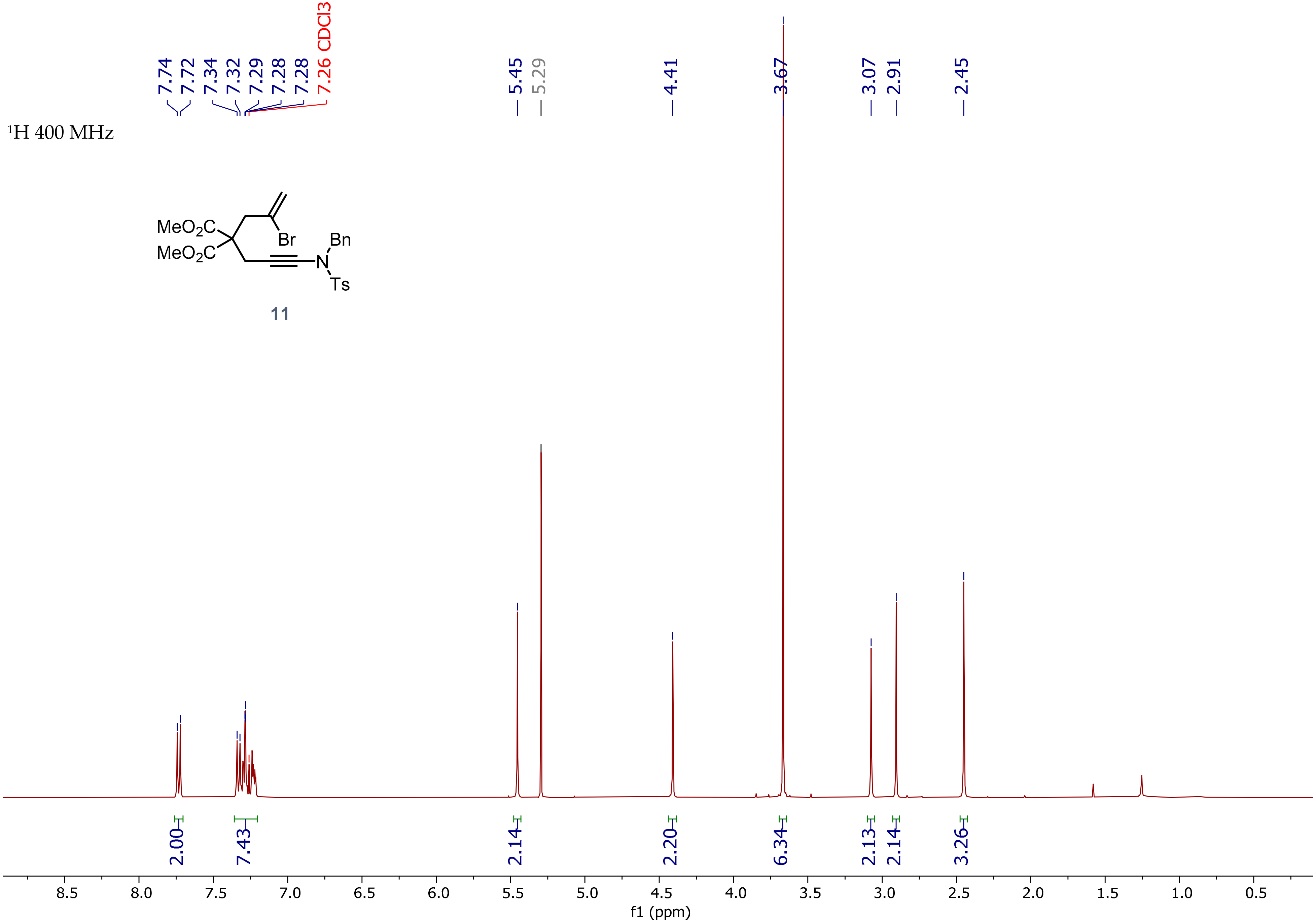

7.74
7.72
7.34
7.32
7.29
7.28
7.28
7.26 CDCl3
5.45
5.29
4.41
3.67
3.07
2.91
2.45
MeO2C
MeO2C
Br
Bn
N
Ts
11
2.00
7.43
2.14
2.20
6.34
2.13
2.14
3.26
8.5
8.0
7.5
7.0
6.5
6.0
5.5
5.0
4.5
4.0
3.5
3.0
2.5
2.0
1.5
1.0
0.5
f1 (ppm)

^{13}C 101 MHz

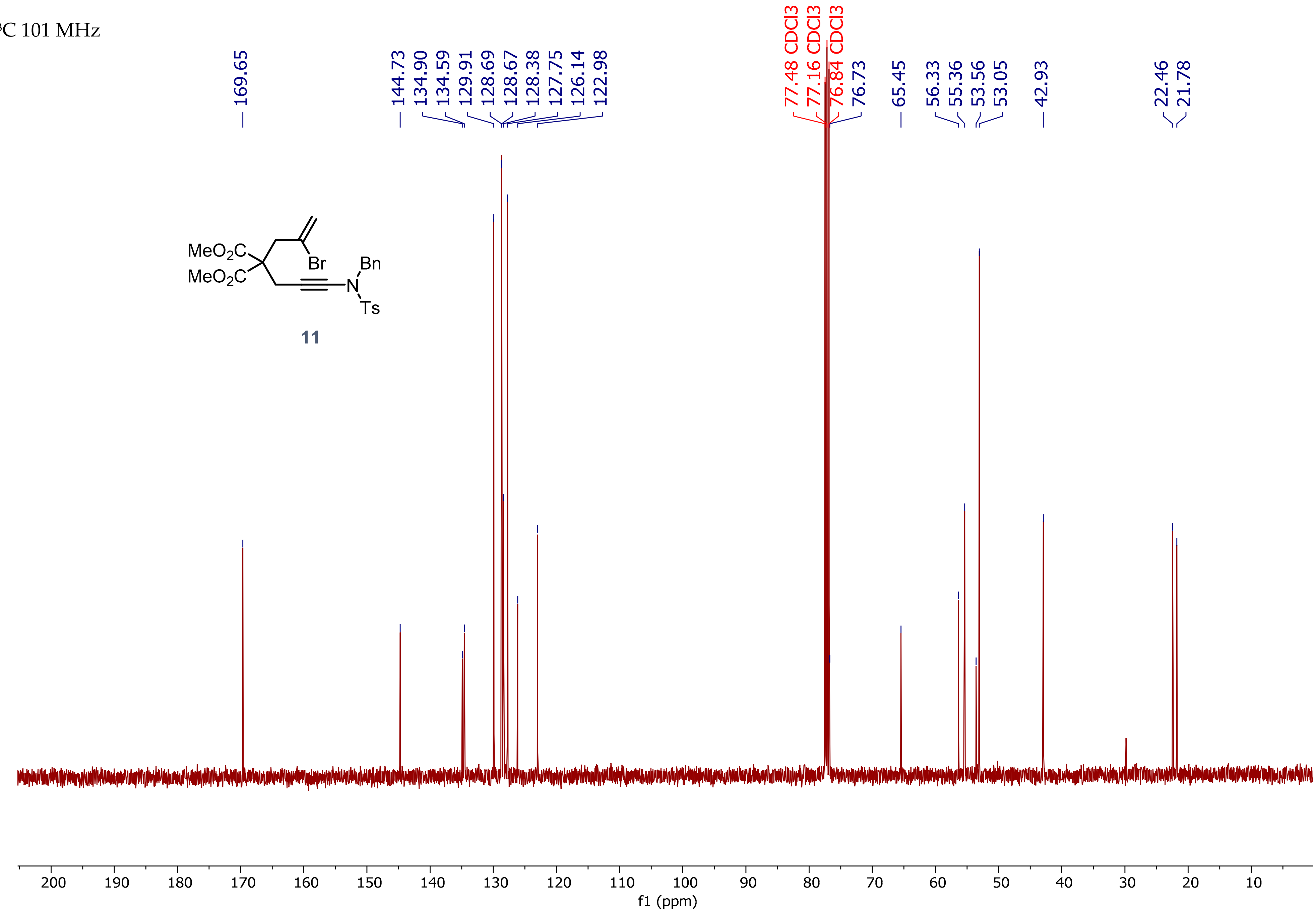
169.65
144.73
134.90
134.59
129.91
128.69
128.67
128.38
127.75
126.14
122.98
77.48 CDCl3
77.16 CDCl3
76.84 CDCl3
76.73
65.45
56.33
55.36
53.56
53.05
42.93
22.46
21.78
MeO2C
MeO2C
Br
Bn
N
Ts
11
200 190 180 170 160 150 140 130 120 110 100 90 80 70 60 50 40 30 20 10
f1 (ppm)

^{1}H 200 MHz

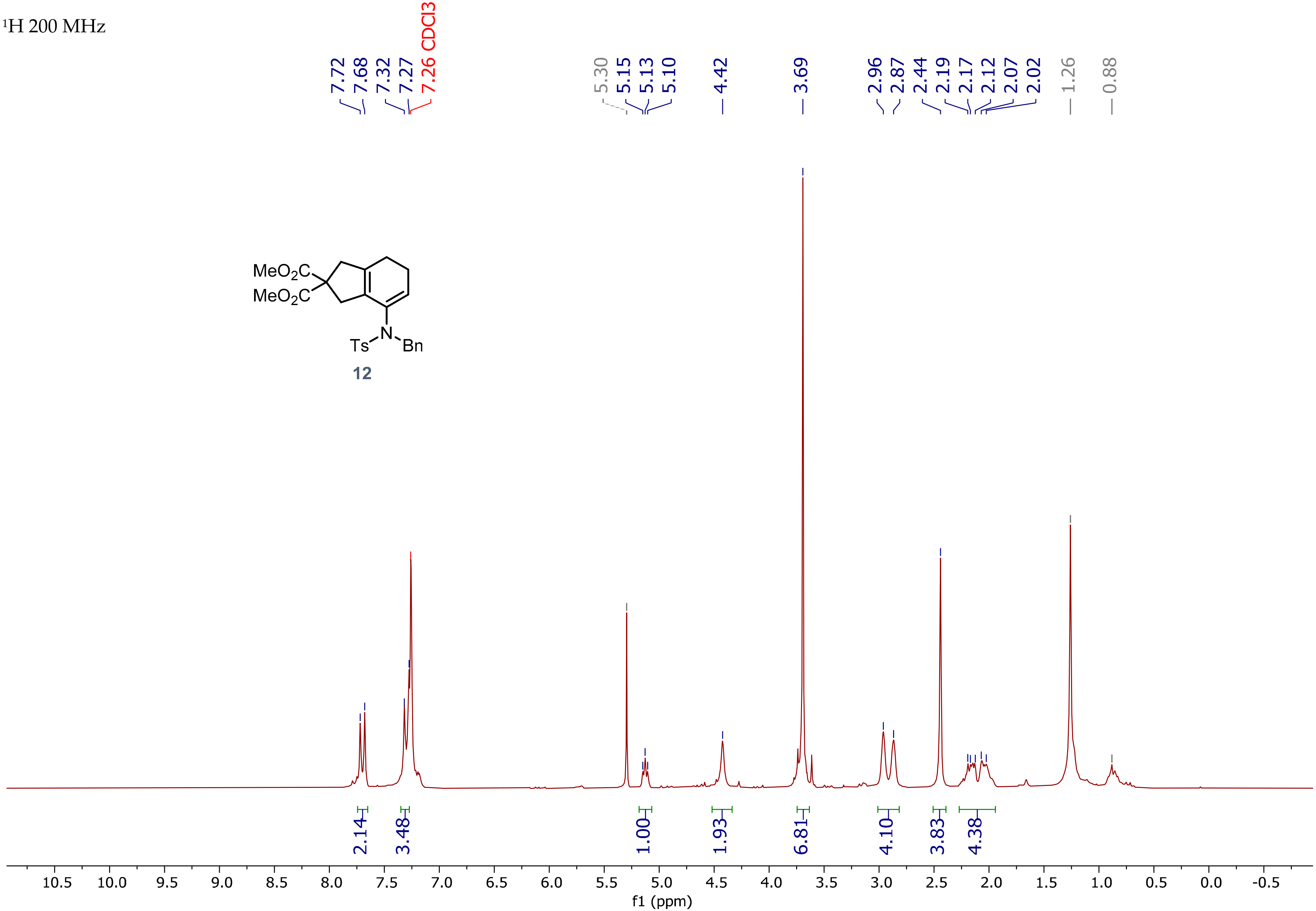
7.72
7.68
7.32
7.27
7.26 CDCl3
5.30
5.15
5.13
5.10
4.42
3.69
2.96
2.87
2.44
2.19
2.17
2.12
2.07
2.02
1.26
0.88
2.14
3.48
1.00
1.93
6.81
4.10
3.83
4.38
10.5 10.0 9.5 9.0 8.5 8.0 7.5 7.0 6.5 6.0 5.5 5.0 4.5 4.0 3.5 3.0 2.5 2.0 1.5 1.0 0.5 0.0 -0.5
f1 (ppm)
MeO2C
MeO2C
Ts
N
Bn

12

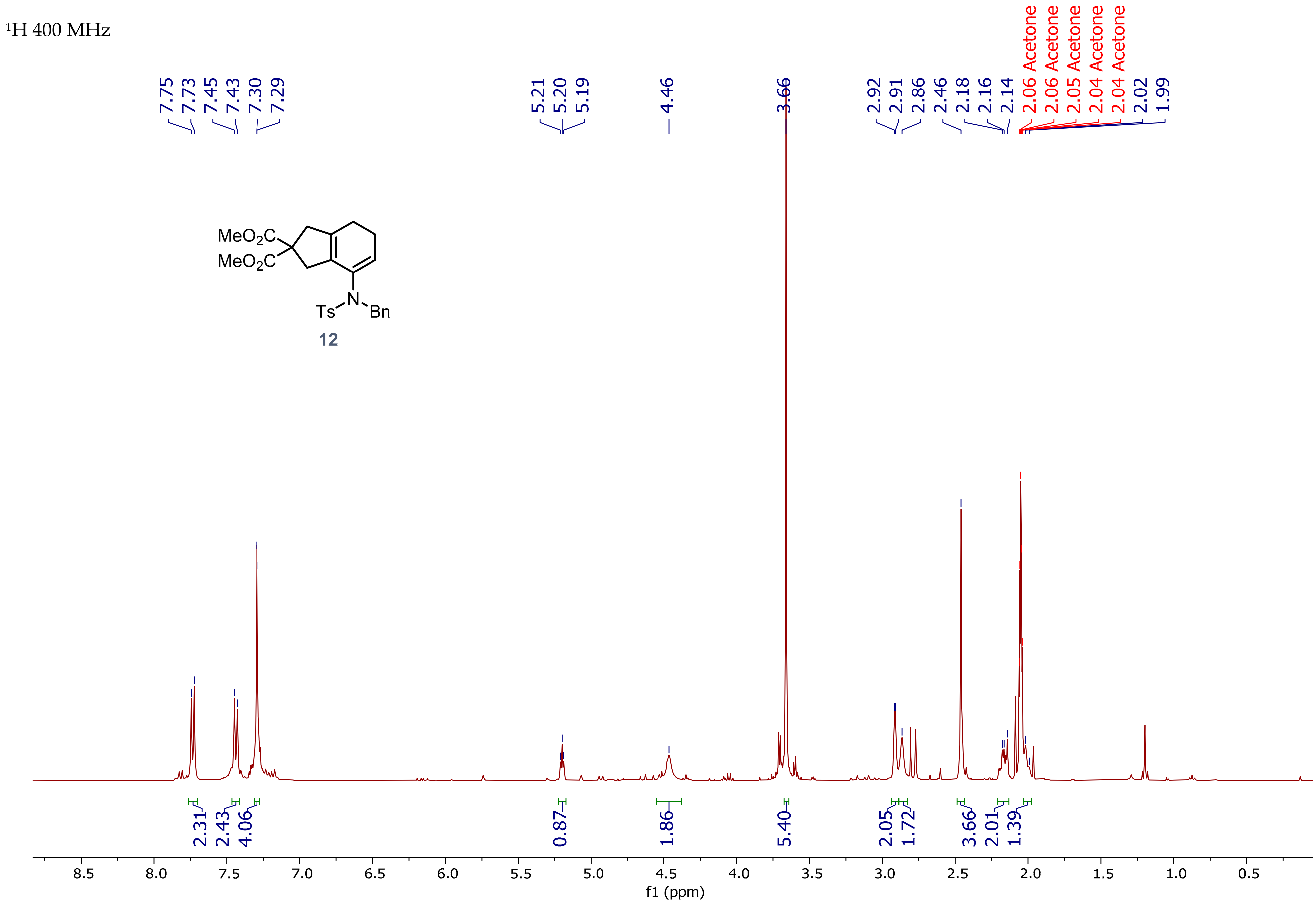

¹H 400 MHz
7.75
7.73
7.45
7.43
7.30
7.29
5.21
5.20
5.19
4.46
3.66
2.92
2.91
2.86
2.46
2.18
2.16
2.14
2.06 Acetone
2.06 Acetone
2.05 Acetone
2.04 Acetone
2.04 Acetone
2.02
1.99
MeO2C
MeO2C
Ts
N
Bn
12
2.31
2.43
4.06
0.87
1.86
5.40
2.05
1.72
3.66
2.01
1.39
8.5
8.0
7.5
7.0
6.5
6.0
5.5
5.0
4.5
4.0
3.5
3.0
2.5
2.0
1.5
1.0
0.5
f1 (ppm)

^{13}C 101 MHz

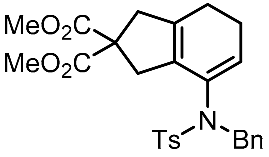

12

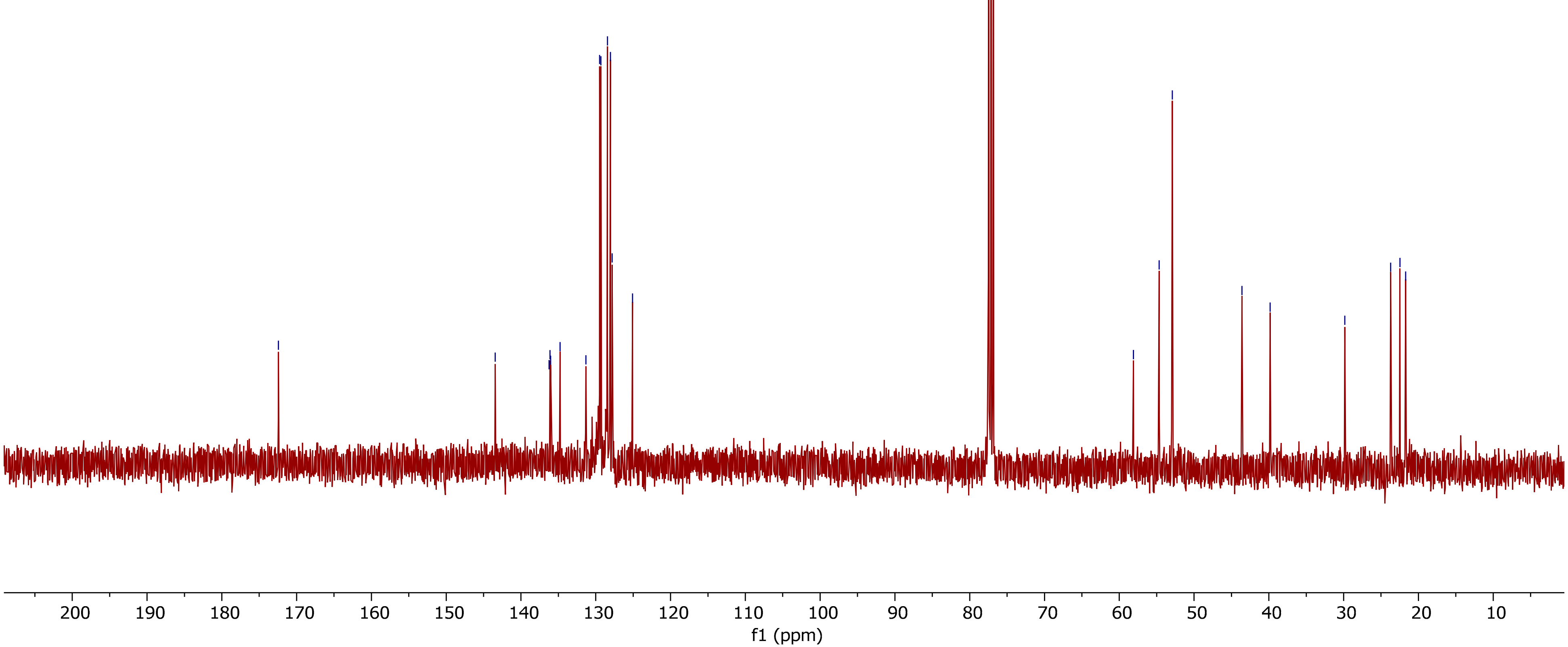

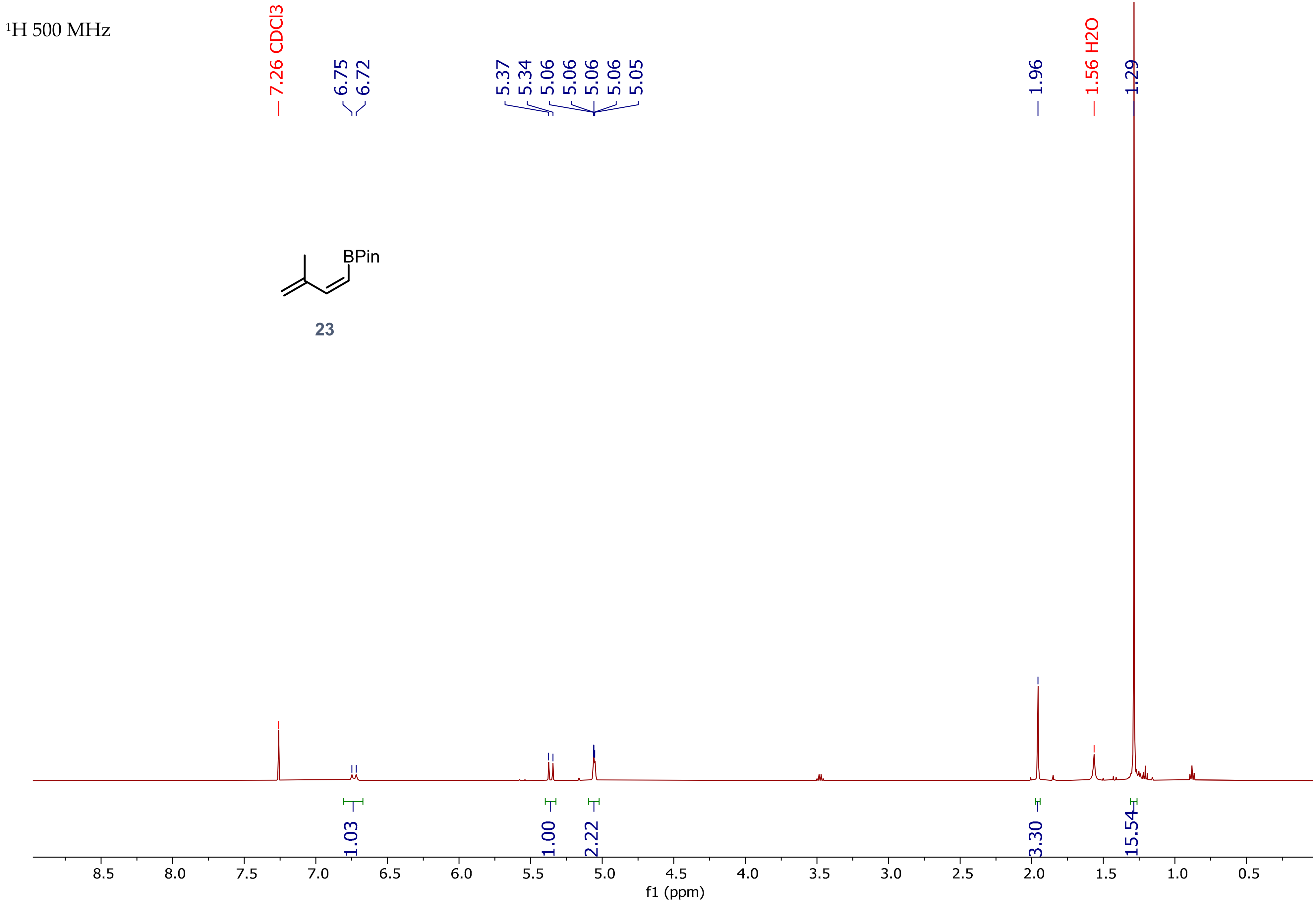

^{1}H 500 MHz
7.26 CDCl3
6.75
6.72
5.37
5.34
5.06
5.06
5.06
5.06
5.05
1.96
1.56 H2O
1.29
BPin
23
1.03
1.00
2.22
3.30
15.54
8.5
8.0
7.5
7.0
6.5
6.0
5.5
5.0
4.5
4.0
3.5
3.0
2.5
2.0
1.5
1.0
0.5
f1 (ppm)

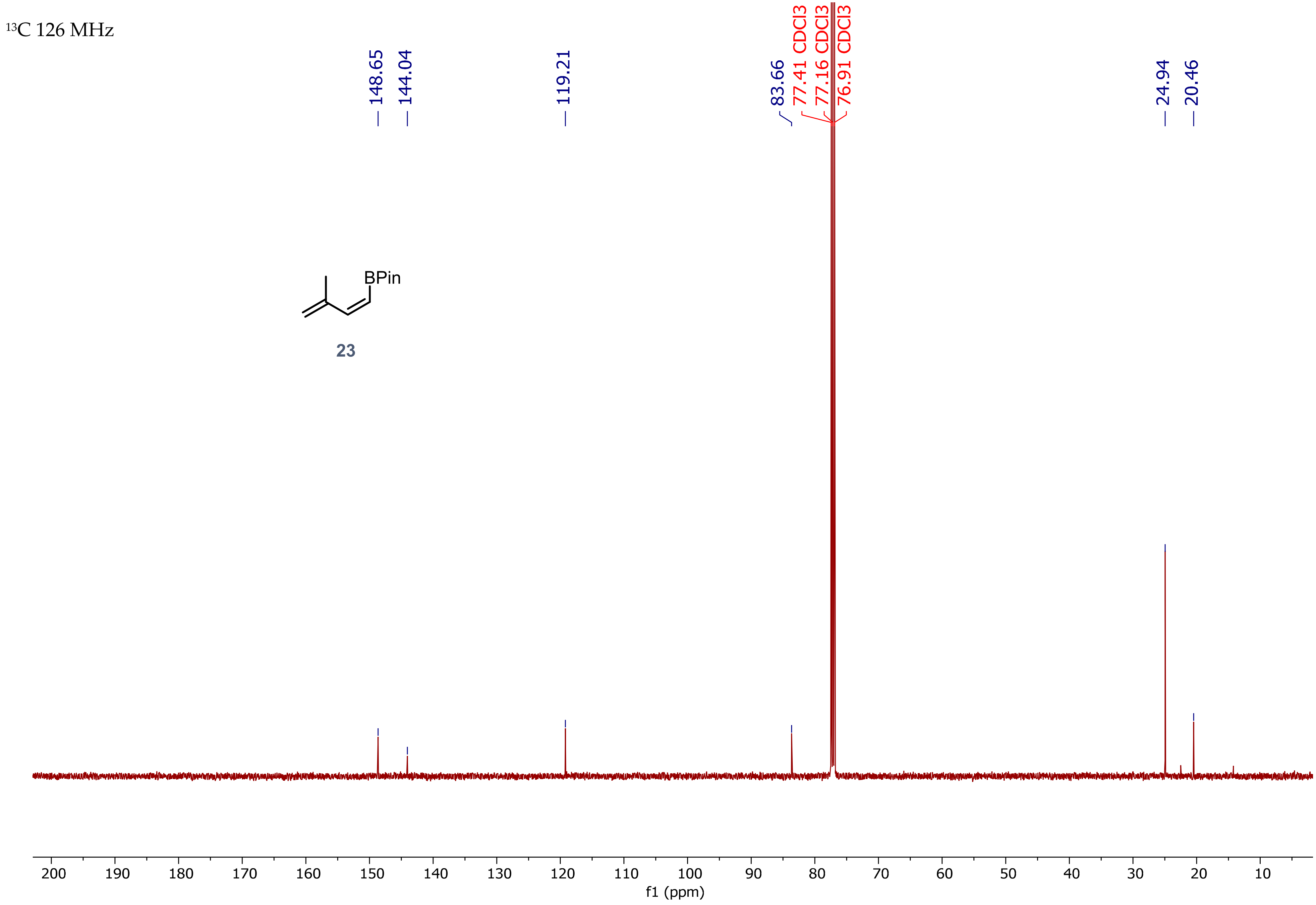

13C 126 MHz
148.65
144.04
119.21
83.66
77.41 CDCl3
77.16 CDCl3
76.91 CDCl3
24.94
20.46
BPin
23
200
190
180
170
160
150
140
130
120
110
100
90
80
70
60
50
40
30
20
10
f1 (ppm)

^{1}H 400 MHz

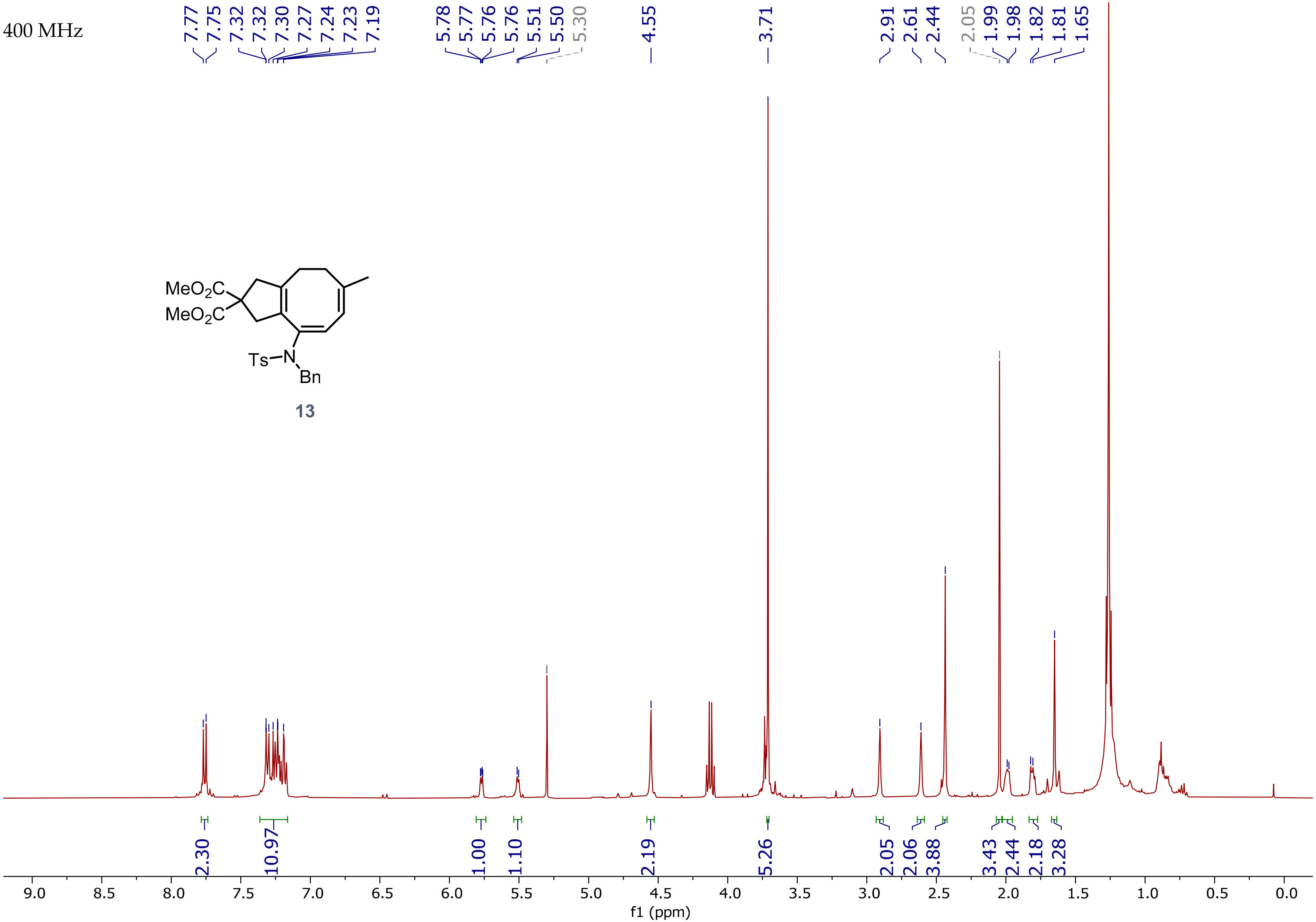
MeO2C
MeO2C
Ts
N
Bn
13
7.77
7.75
7.32
7.32
7.30
7.27
7.24
7.23
7.19
5.78
5.77
5.76
5.76
5.51
5.50
5.30
4.55
3.71
2.91
2.61
2.44
2.05
1.99
1.98
1.82
1.81
1.65
2.30
10.97
1.00
1.10
2.19
5.26
2.05
2.06
3.88
3.43
2.44
2.18
3.28
9.0
8.5
8.0
7.5
7.0
6.5
6.0
5.5
5.0
4.5
4.0
3.5
3.0
2.5
2.0
1.5
1.0
0.5
0.0
f1 (ppm)

^{13}C 101 MHz

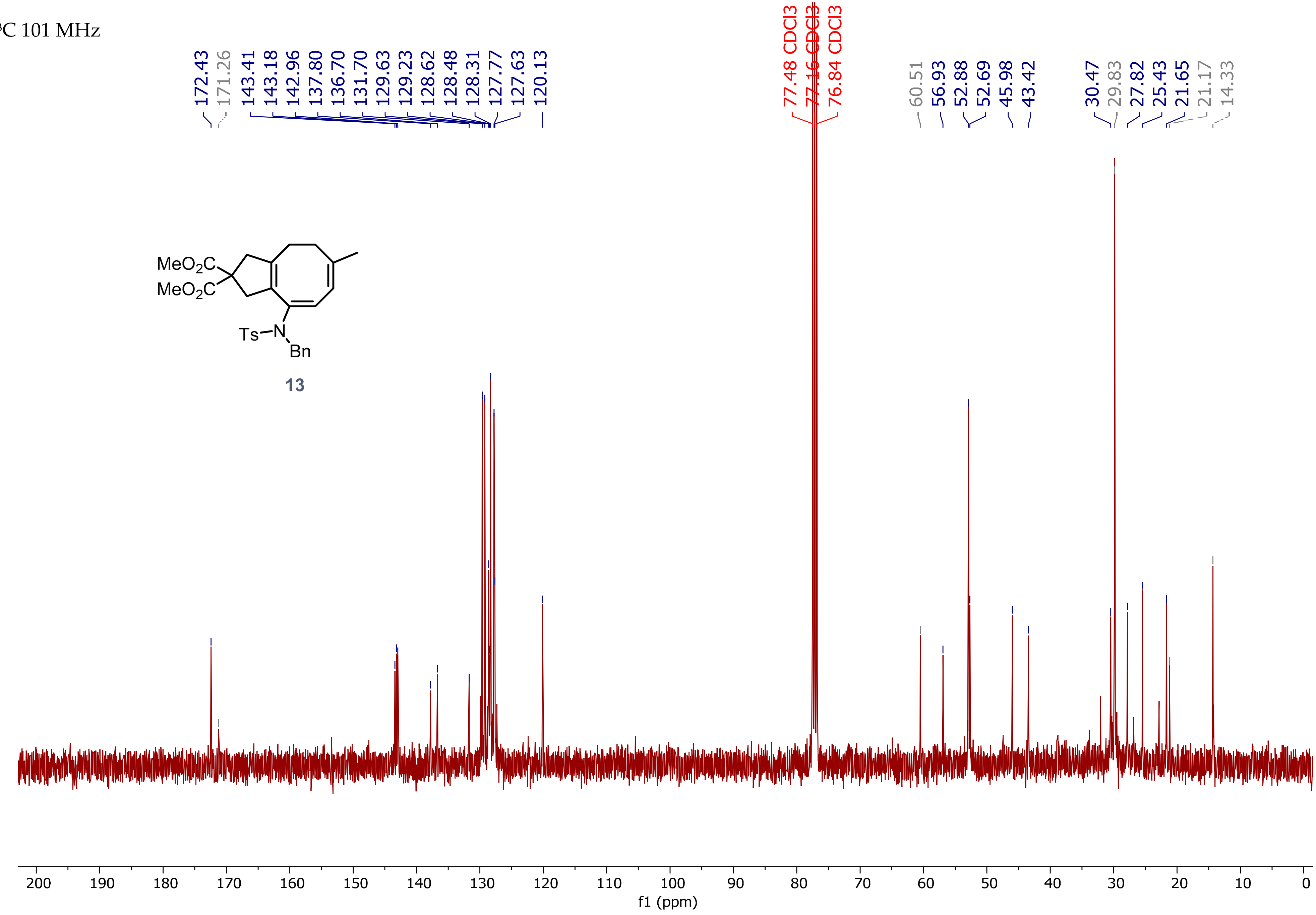

172.43
171.26
143.41
143.18
142.96
137.80
136.70
131.70
129.63
129.23
128.62
128.48
128.31
127.77
127.63
120.13
77.48 CDCl3
77.16 CDCl3
76.84 CDCl3
60.51
56.93
52.88
52.69
45.98
43.42
30.47
29.83
27.82
25.43
21.65
21.17
14.33
MeO2C
MeO2C
Ts
N
Bn
13
200
190
180
170
160
150
140
130
120
110
100
90
80
70
60
50
40
30
20
10
0
f1 (ppm)

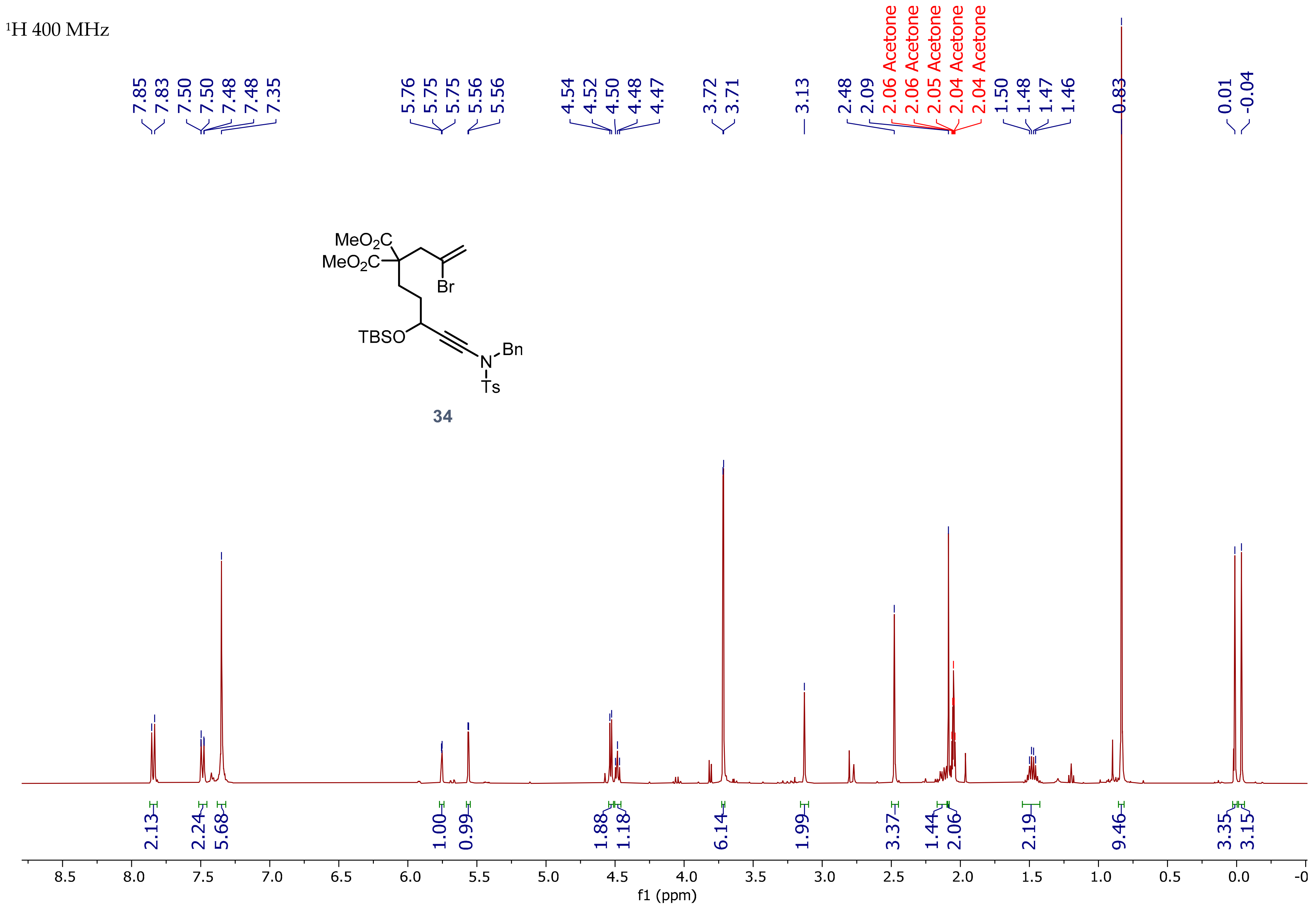

¹H 400 MHz
7.85
7.83
7.50
7.50
7.48
7.48
7.35
5.76
5.75
5.75
5.56
5.56
4.54
4.52
4.50
4.48
4.47
3.72
3.71
3.13
2.48
2.09
2.06 Acetone
2.06 Acetone
2.05 Acetone
2.04 Acetone
2.04 Acetone
1.50
1.48
1.47
1.46
0.83
0.01
-0.04
MeO2C
MeO2C
Br
TBSO
N
Bn
Ts
34
2.13
2.24
5.68
1.00
0.99
1.88
1.18
6.14
1.99
3.37
1.44
2.06
2.19
9.46
3.35
3.15
8.5
8.0
7.5
7.0
6.5
6.0
5.5
5.0
4.5
4.0
3.5
3.0
2.5
2.0
1.5
1.0
0.5
0.0
-0
f1 (ppm)

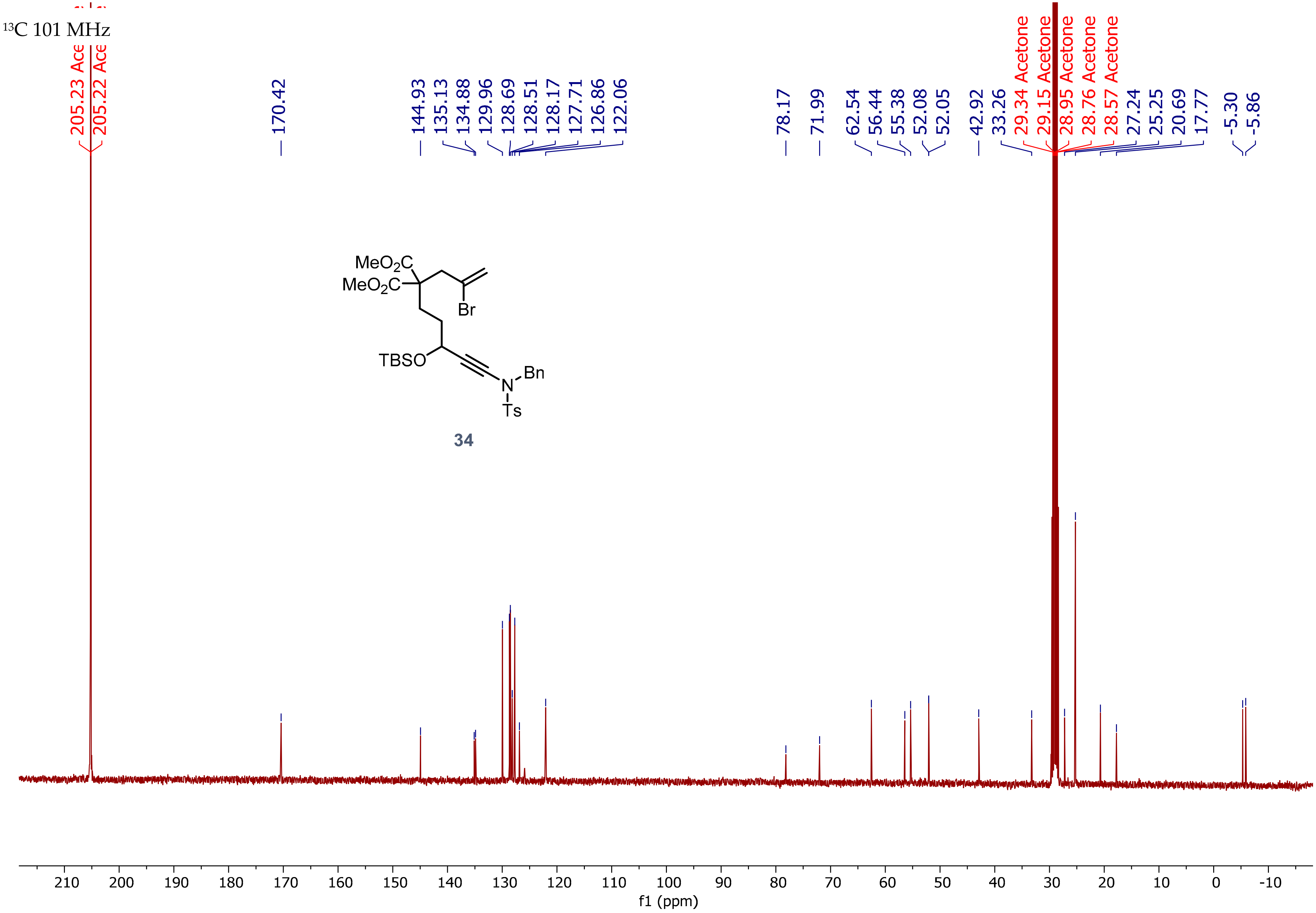

13C 101 MHz
205.23 Acetone
205.22 Acetone
170.42
144.93
135.13
134.88
129.96
128.69
128.51
128.17
127.71
126.86
122.06
78.17
71.99
62.54
56.44
55.38
52.08
52.05
42.92
33.26
29.34 Acetone
29.15 Acetone
28.95 Acetone
28.76 Acetone
28.57 Acetone
27.24
25.25
20.69
17.77
-5.30
-5.86
MeO2C
MeO2C
Br
TBSO
N
Bn
Ts
34
210
200
190
180
170
160
150
140
130
120
110
100
90
80
70
60
50
40
30
20
10
0
-10
f1 (ppm)

^{1}H 400 MHz

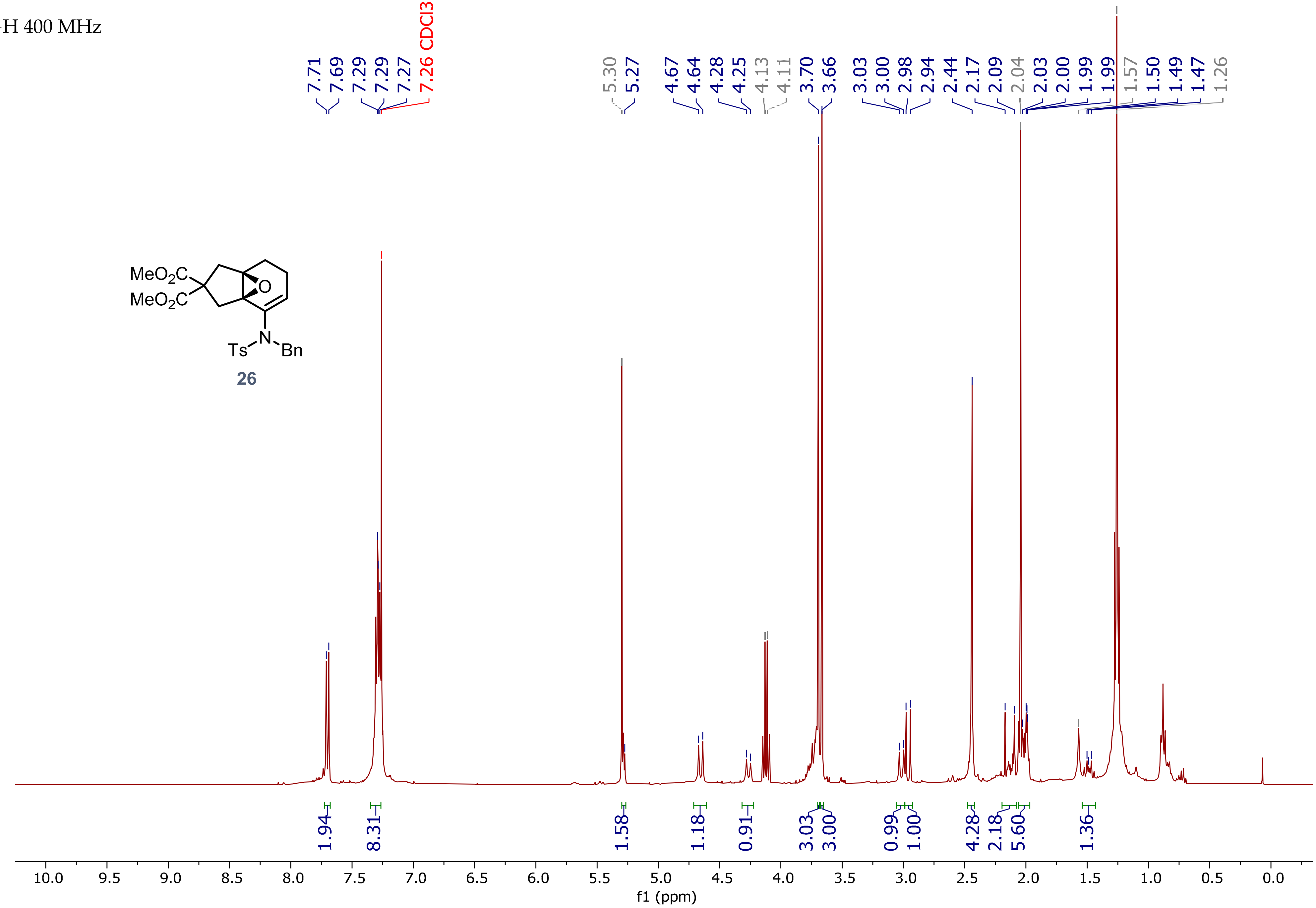

26
7.71
7.69
7.29
7.29
7.27
7.26 CDCl3
5.30
5.27
4.67
4.64
4.28
4.25
4.13
4.11
3.70
3.66
3.03
3.00
2.98
2.94
2.44
2.17
2.09
2.04
2.03
2.00
1.99
1.99
1.57
1.50
1.49
1.47
1.26
1.94
8.31
1.58
1.18
0.91
3.03
3.00
0.99
1.00
4.28
2.18
5.60
1.36
10.0
9.5
9.0
8.5
8.0
7.5
7.0
6.5
6.0
5.5
5.0
4.5
4.0
3.5
3.0
2.5
2.0
1.5
1.0
0.5
0.0
f1 (ppm)

^{13}C 126 MHz

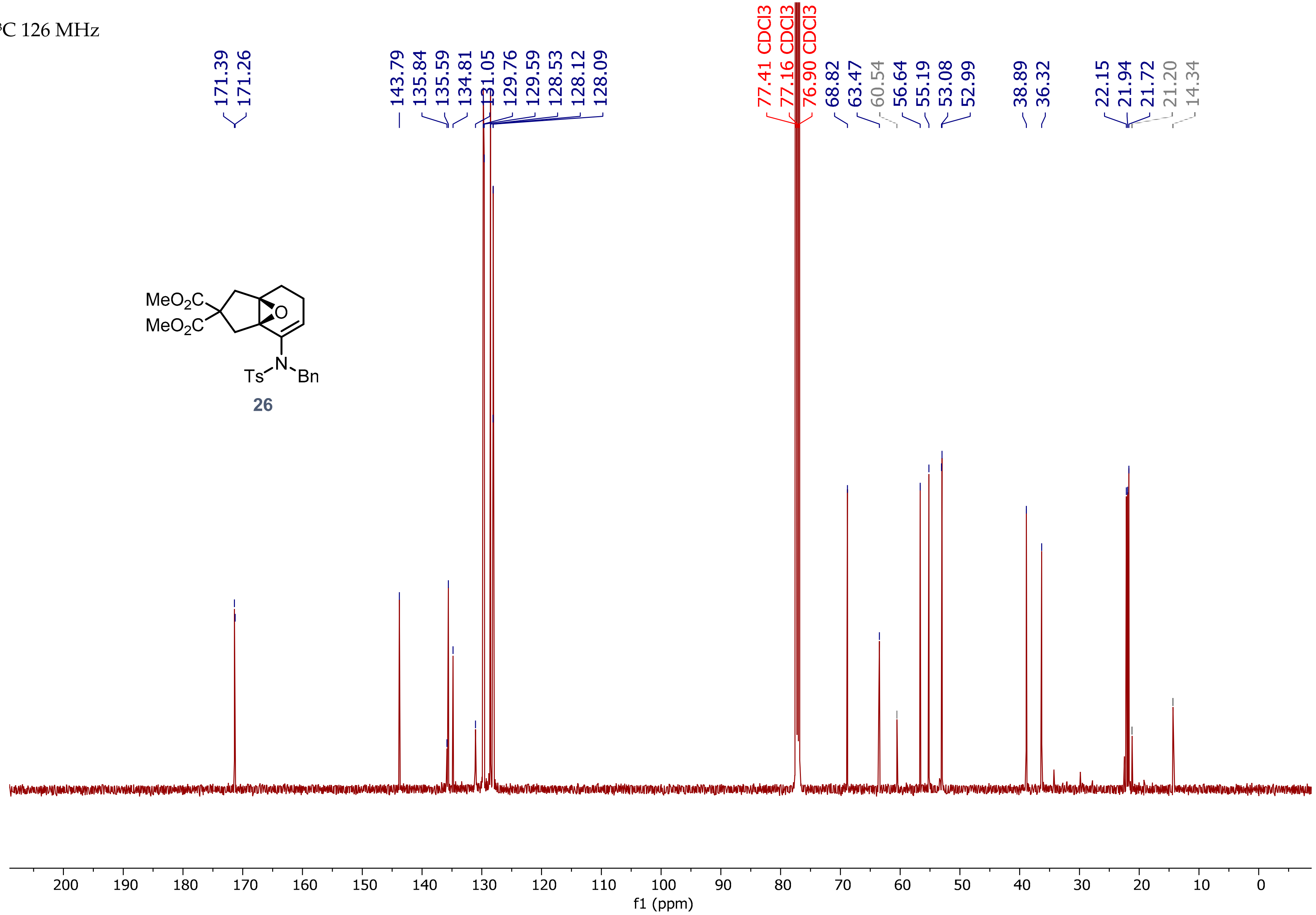

171.39
171.26
143.79
135.84
135.59
134.81
131.05
129.76
129.59
128.53
128.12
128.09
77.41 CDCl3
77.16 CDCl3
76.90 CDCl3
68.82
63.47
60.54
56.64
55.19
53.08
52.99
38.89
36.32
22.15
21.94
21.72
21.20
14.34
MeO2C
MeO2C
O
N
Ts
Bn
26
200 190 180 170 160 150 140 130 120 110 100 90 80 70 60 50 40 30 20 10 0
f1 (ppm)

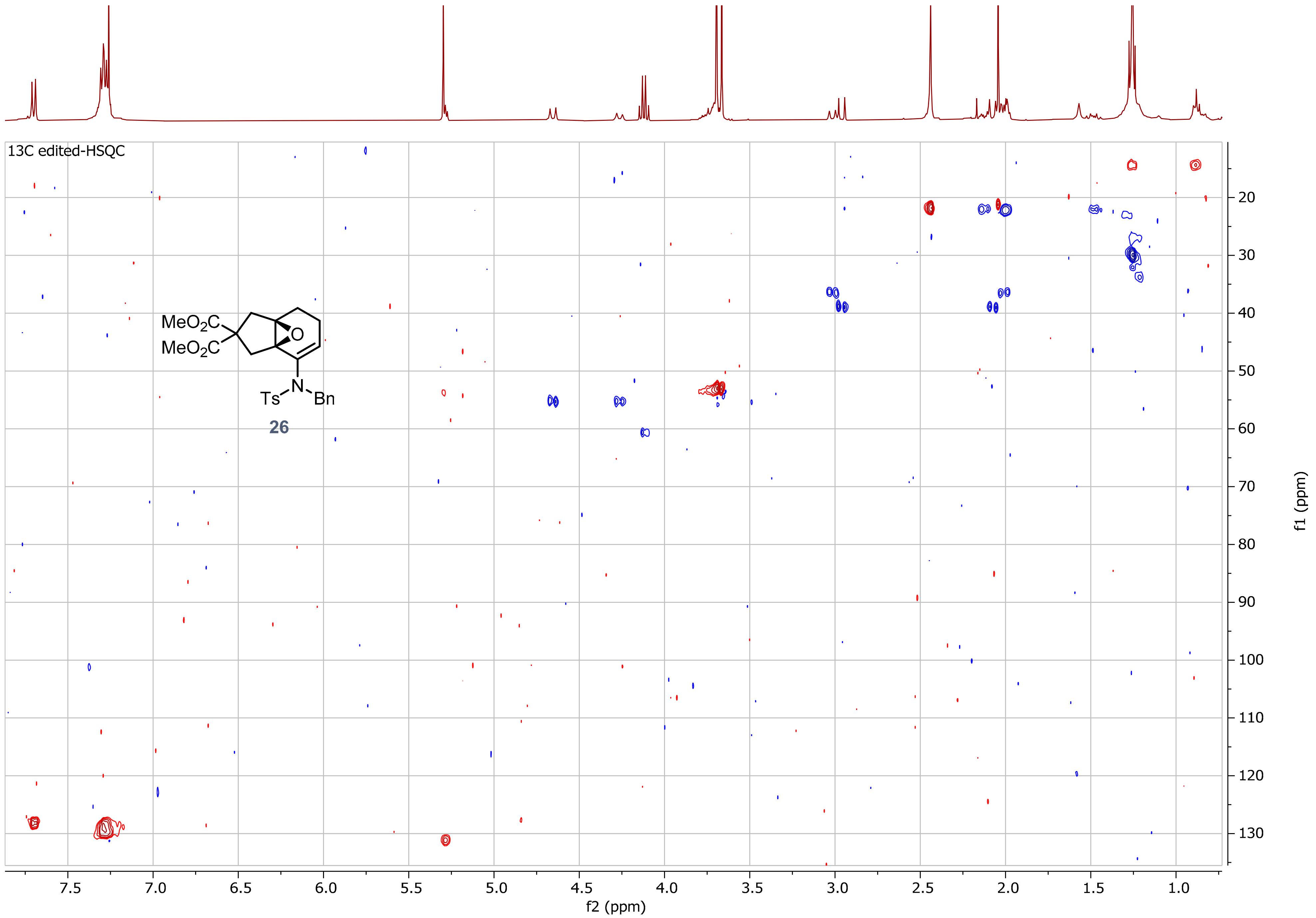
13C edited-HSQC
MeO2C
MeO2C
O
N
Ts
Bn
26
7.5
7.0
6.5
6.0
5.5
5.0
4.5
4.0
3.5
3.0
2.5
2.0
1.5
1.0
f2 (ppm)
20
30
40
50
60
70
80
90
100
110
120
130
f1 (ppm)

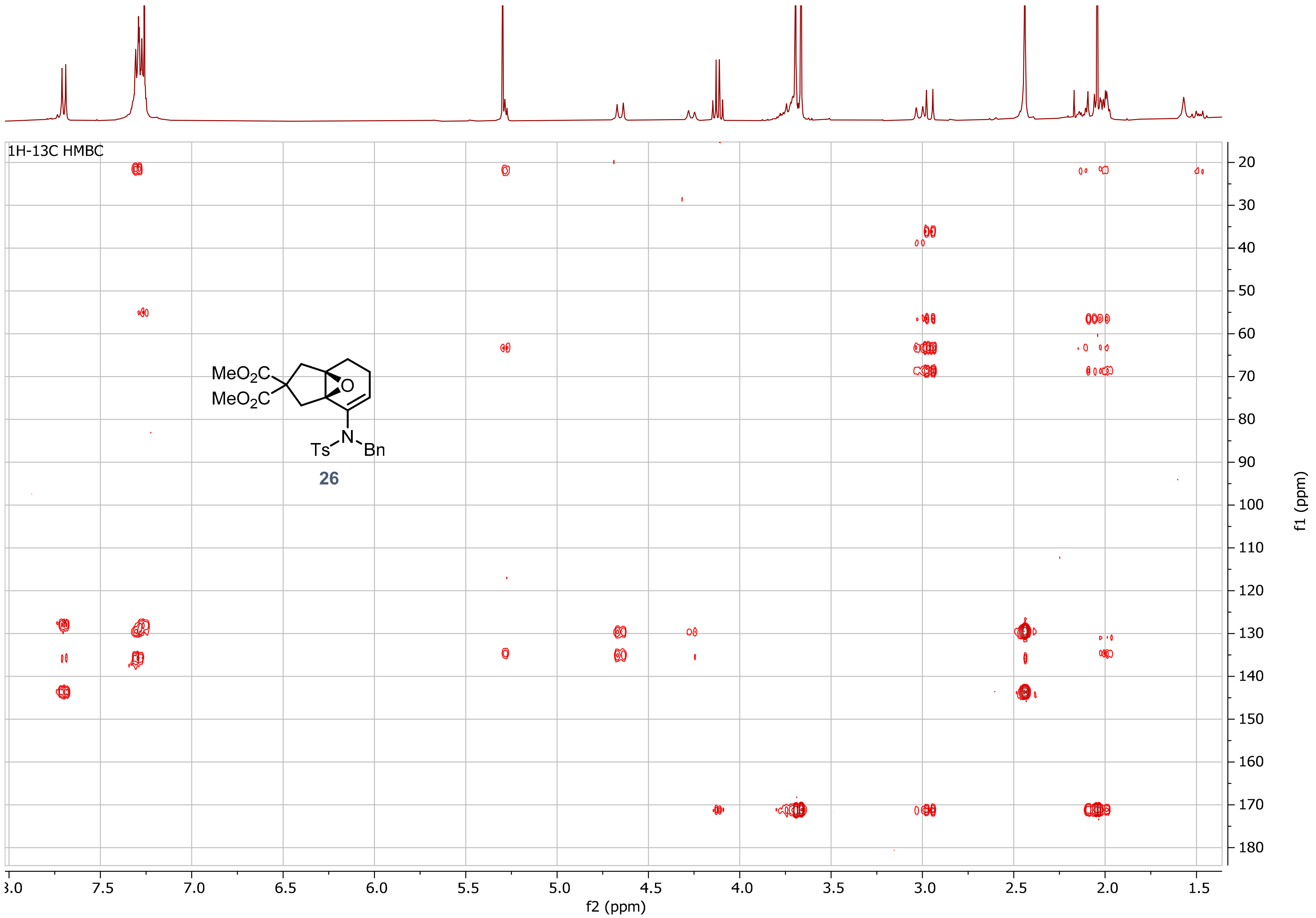
1H-13C HMBC
MeO2C
MeO2C
O
N
Ts
Bn
26
f1 (ppm)
f2 (ppm)

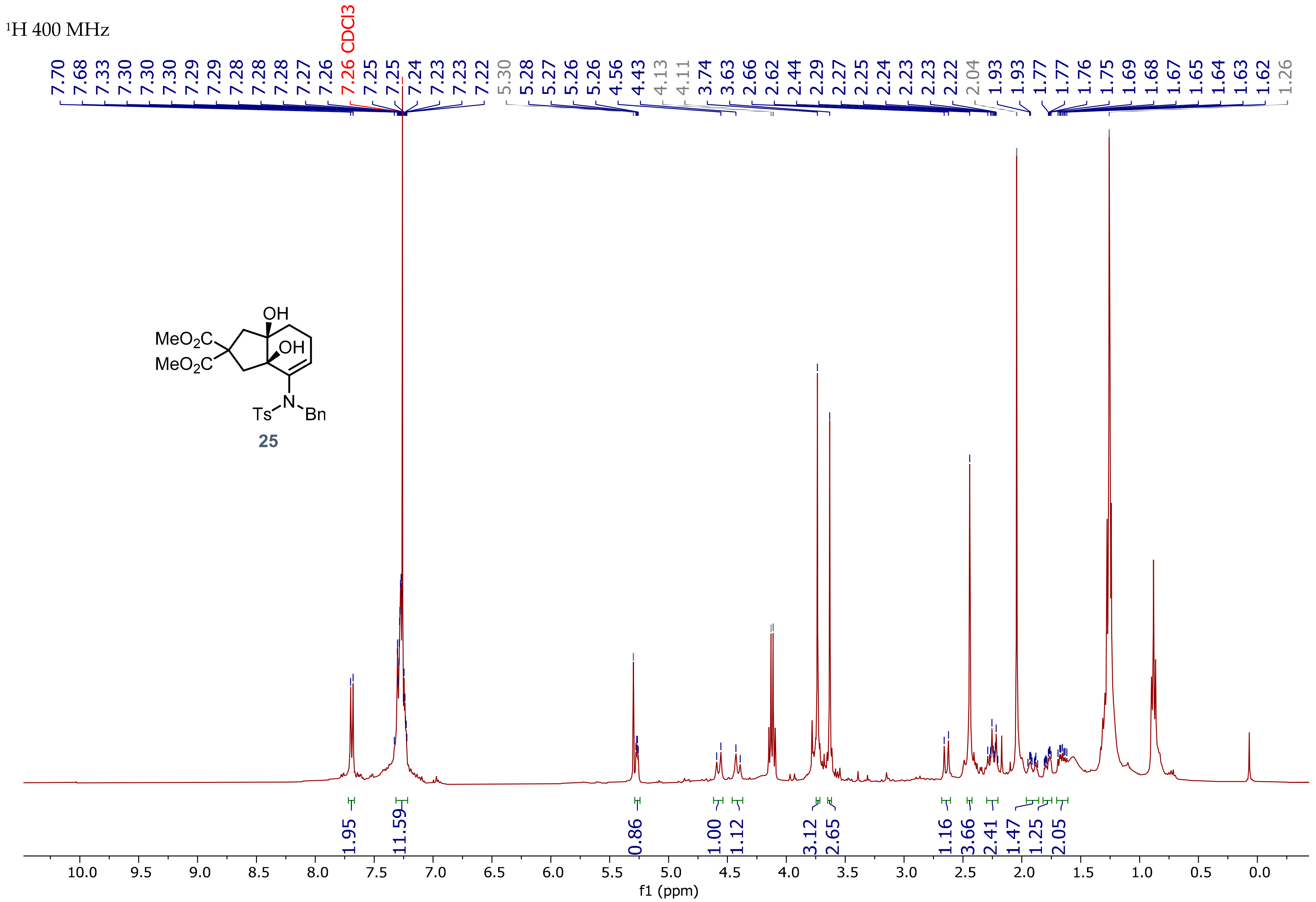

¹H 400 MHz
7.70 7.68 7.33 7.30 7.30 7.30 7.29 7.29 7.28 7.28 7.28 7.27 7.26 7.26 CDCl3 7.25 7.25 7.24 7.23 7.23 7.22 5.30 5.28 5.27 5.26 5.26 4.56 4.43 4.13 4.11 3.74 3.63 2.66 2.62 2.44 2.29 2.27 2.25 2.24 2.23 2.23 2.22 2.04 1.93 1.93 1.77 1.77 1.76 1.75 1.69 1.68 1.67 1.65 1.64 1.63 1.62 1.26
MeO2C
MeO2C
OH
OH
Ts
N
Bn
25
1.95 11.59 0.86 1.00 1.12 3.12 2.65 1.16 3.66 2.41 1.47 1.25 2.05
10.0 9.5 9.0 8.5 8.0 7.5 7.0 6.5 6.0 5.5 5.0 4.5 4.0 3.5 3.0 2.5 2.0 1.5 1.0 0.5 0.0
f1 (ppm)

^{13}C 126 MHz

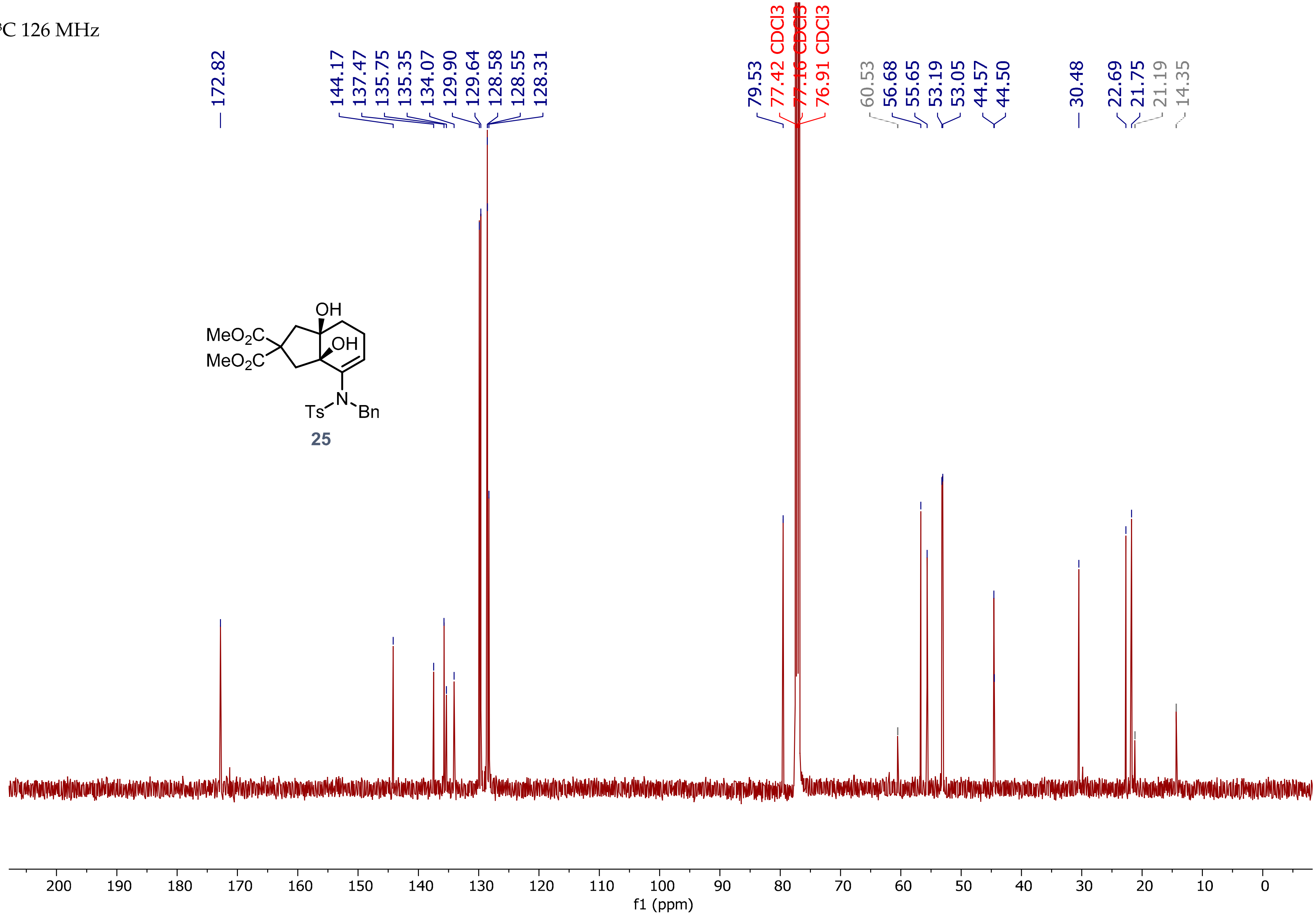

172.82
144.17
137.47
135.75
135.35
134.07
129.90
129.64
128.58
128.55
128.31
79.53
77.42 CDCl3
77.16 CDCl3
76.91 CDCl3
60.53
56.68
55.65
53.19
53.05
44.57
44.50
30.48
22.69
21.75
21.19
14.35
MeO2C
MeO2C
OH
OH
Ts
N
Bn
25
200 190 180 170 160 150 140 130 120 110 100 90 80 70 60 50 40 30 20 10 0
f1 (ppm)

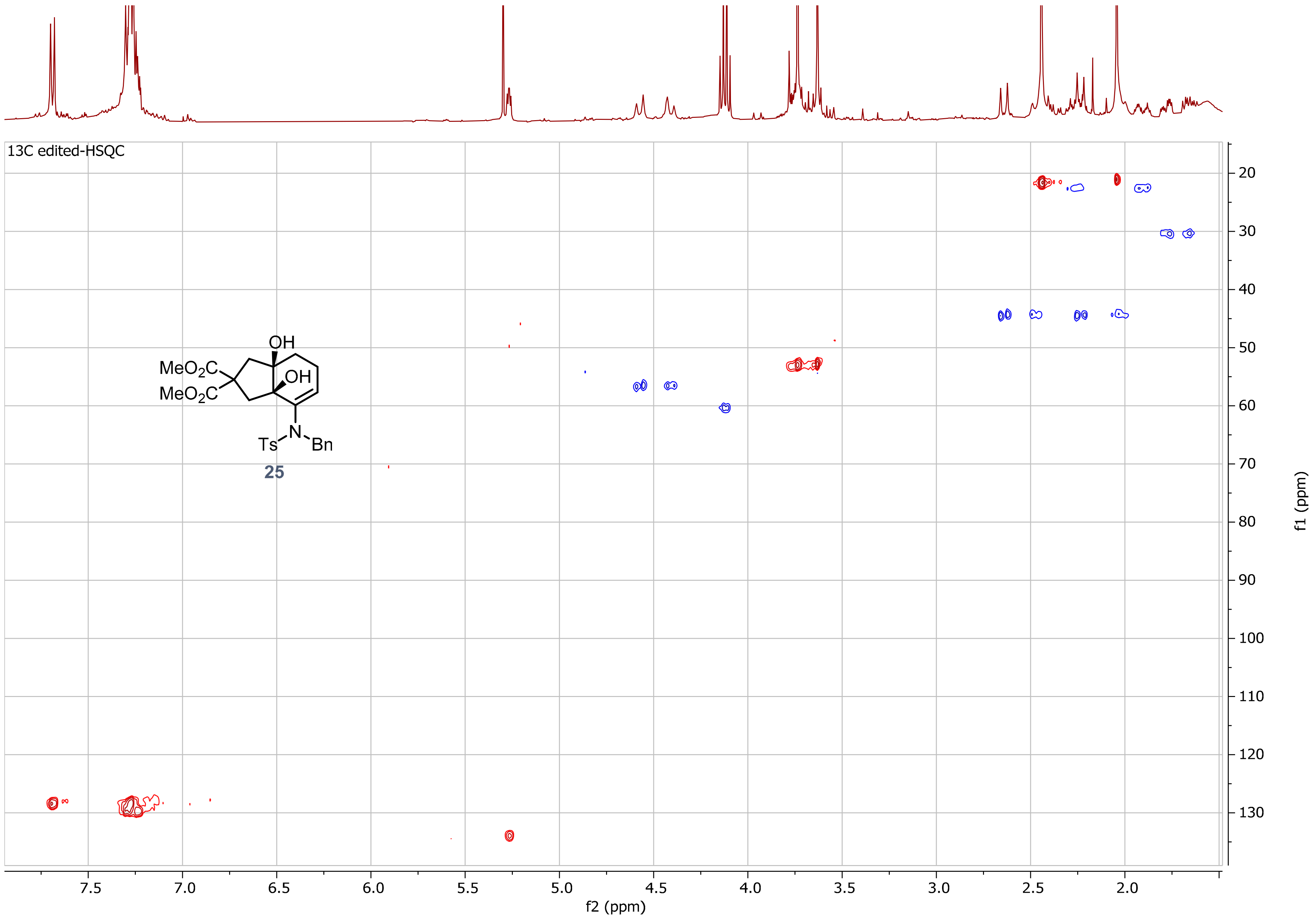
13C edited-HSQC
MeO2C
MeO2C
OH
OH
Ts
N
Bn
25
f2 (ppm)
f1 (ppm)

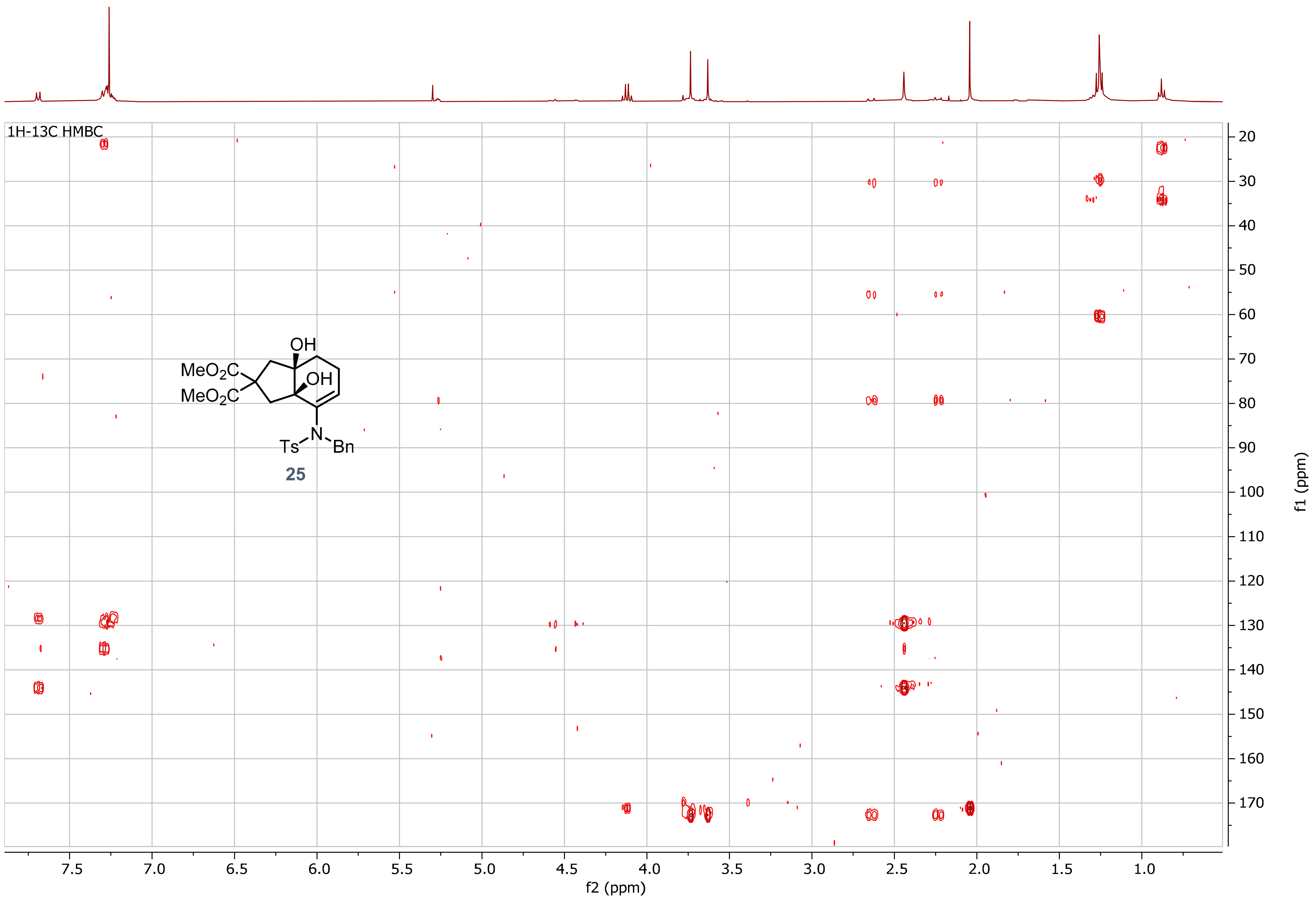

1H-13C HMBC
MeO2C
MeO2C
OH
OH
N
Ts
Bn
25
f2 (ppm)
f1 (ppm)

^{1}H 400 MHz

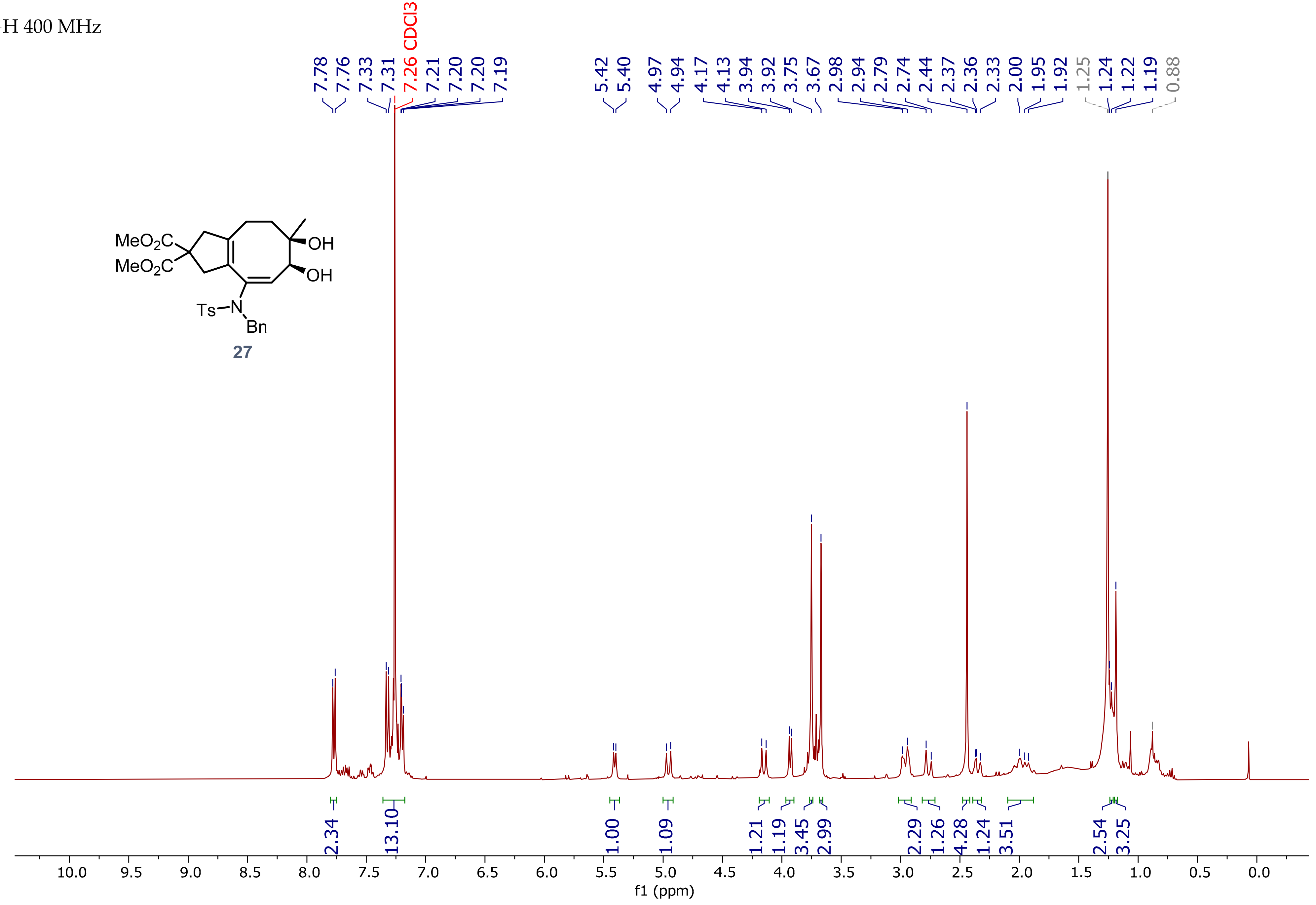
7.78
7.76
7.33
7.31
7.26 CDCl3
7.21
7.20
7.20
7.19
5.42
5.40
4.97
4.94
4.17
4.13
3.94
3.92
3.75
3.67
2.98
2.94
2.79
2.74
2.44
2.37
2.36
2.33
2.00
1.95
1.92
1.25
1.24
1.22
1.19
0.88
MeO2C
MeO2C
OH
OH
Ts
N
Bn
27
2.34
13.10
1.00
1.09
1.21
1.19
3.45
2.99
2.29
1.26
4.28
1.24
3.51
2.54
3.25
10.0
9.5
9.0
8.5
8.0
7.5
7.0
6.5
6.0
5.5
5.0
4.5
4.0
3.5
3.0
2.5
2.0
1.5
1.0
0.5
0.0
f1 (ppm)

^{13}C 126 MHz

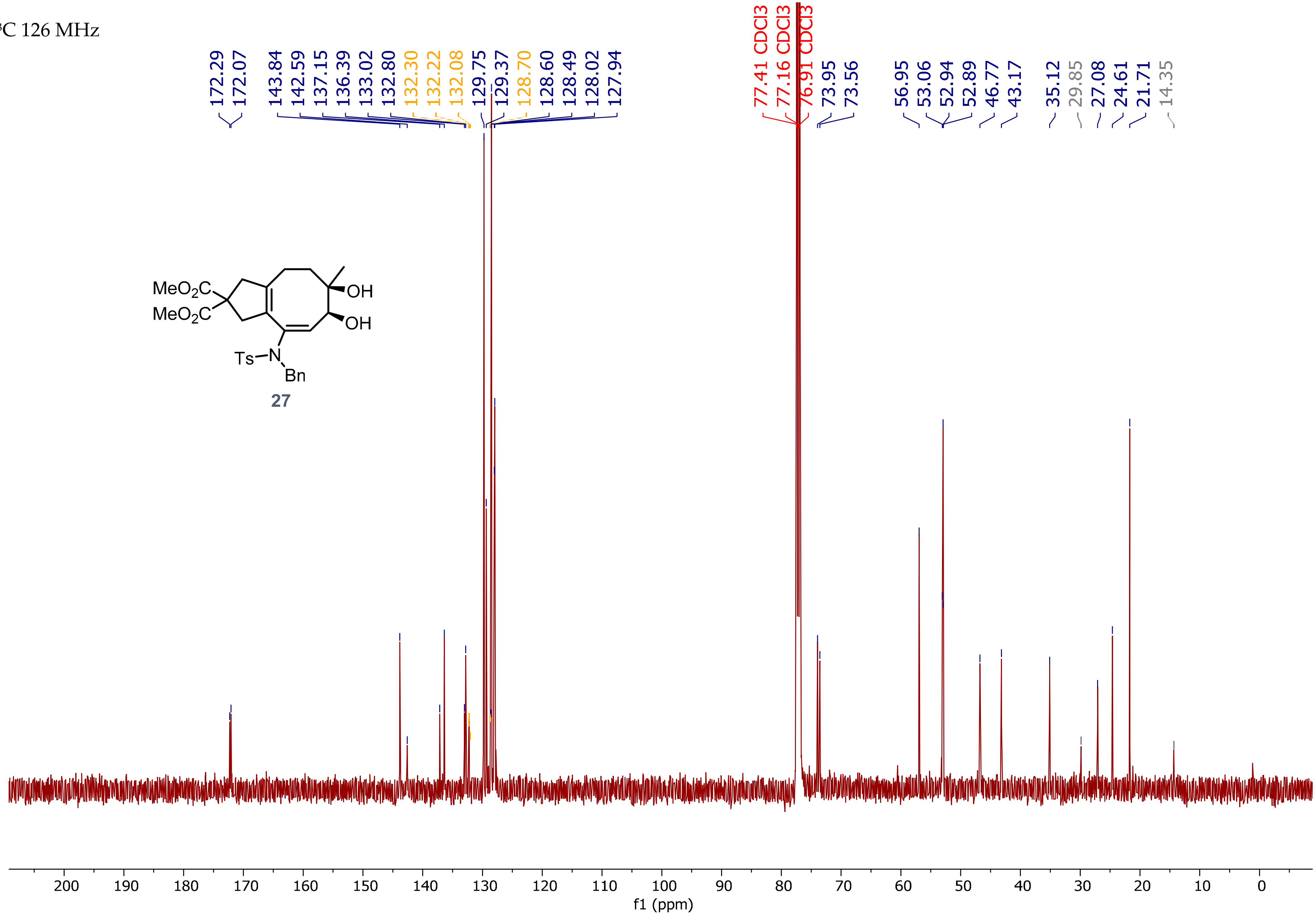
172.29
172.07
143.84
142.59
137.15
136.39
133.02
132.80
132.30
132.22
132.08
129.75
129.37
128.70
128.60
128.49
128.02
127.94
77.41 CDCl3
77.16 CDCl3
76.91 CDCl3
73.95
73.56
56.95
53.06
52.94
52.89
46.77
43.17
35.12
29.85
27.08
24.61
21.71
14.35
MeO2C
MeO2C
OH
OH
Ts
N
Bn
27
200
190
180
170
160
150
140
130
120
110
100
90
80
70
60
50
40
30
20
10
0
f1 (ppm)

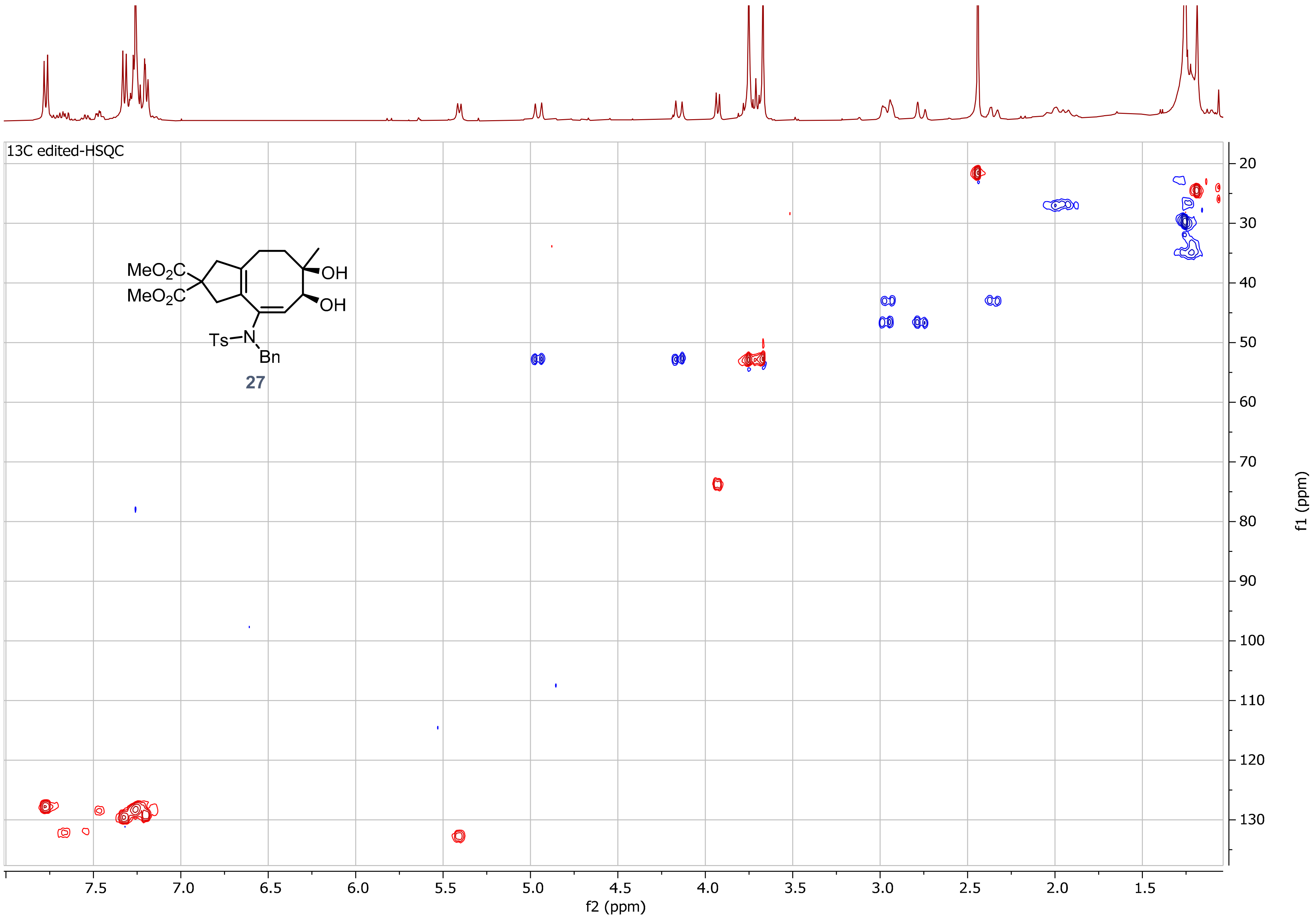
13C edited-HSQC
MeO2C
MeO2C
OH
OH
Ts
N
Bn
27
f2 (ppm)
f1 (ppm)

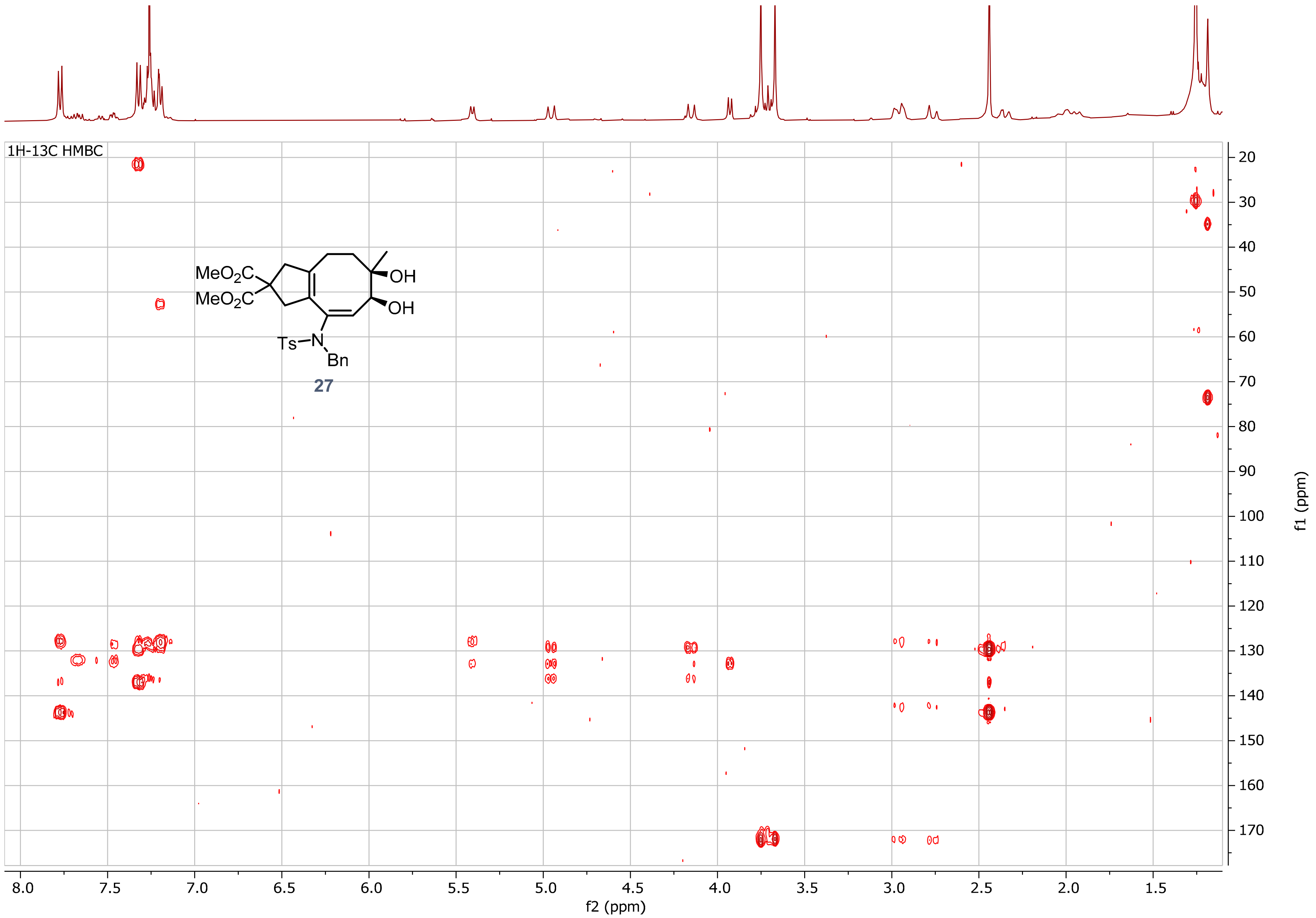
1H-13C HMBC
MeO2C
MeO2C
OH
OH
Ts
N
Bn
27
f2 (ppm)
f1 (ppm)
8.0 7.5 7.0 6.5 6.0 5.5 5.0 4.5 4.0 3.5 3.0 2.5 2.0 1.5
20 30 40 50 60 70 80 90 100 110 120 130 140 150 160 170

^{1}H 400 MHz

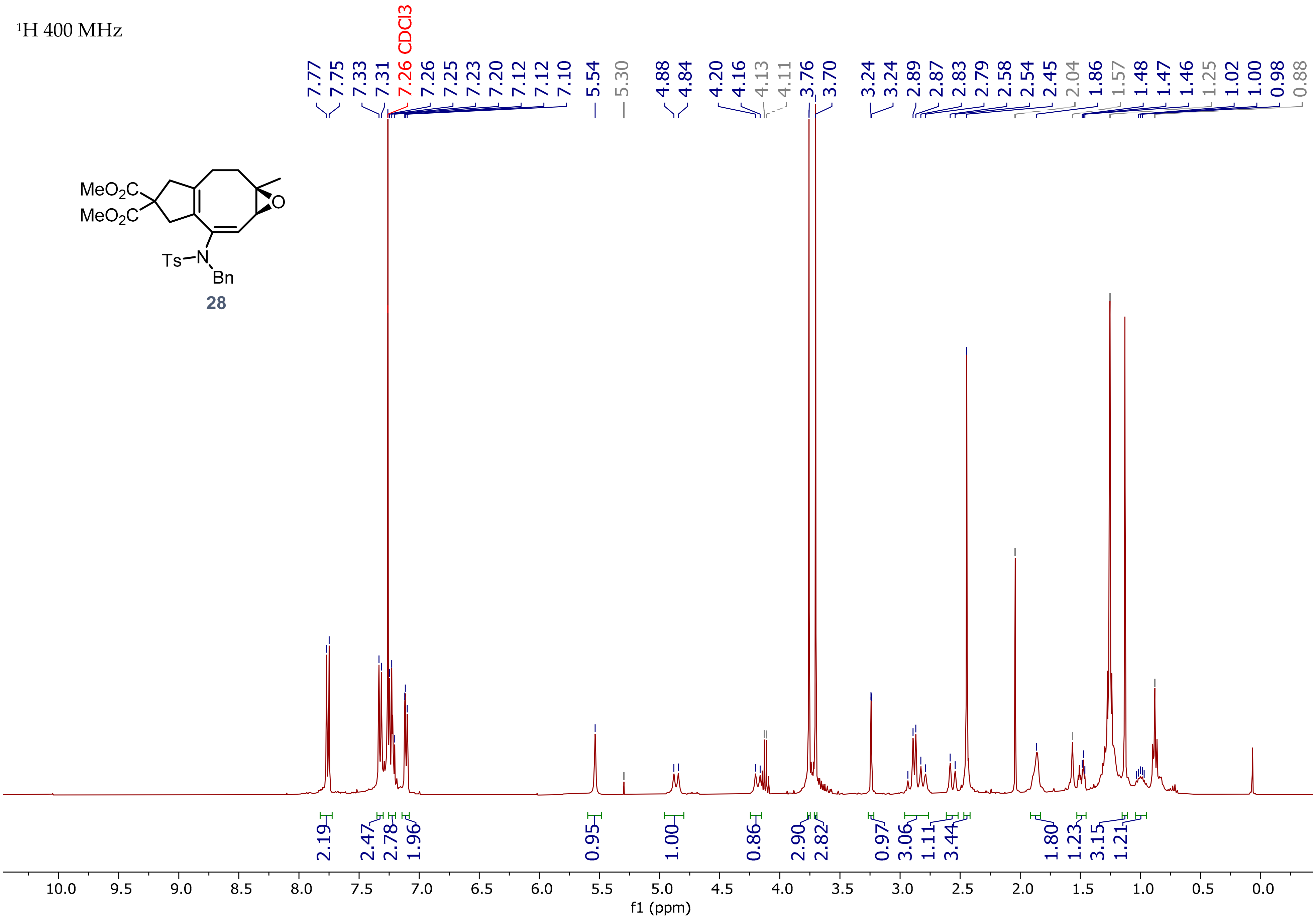

MeO2C
MeO2C
O
Ts
N
Bn
28
7.77
7.75
7.33
7.31
7.26 CDCl3
7.26
7.25
7.23
7.20
7.12
7.12
7.10
5.54
5.30
4.88
4.84
4.20
4.16
4.13
4.11
3.76
3.70
3.24
3.24
2.89
2.87
2.83
2.79
2.58
2.54
2.45
2.04
1.86
1.57
1.48
1.47
1.46
1.25
1.02
1.00
0.98
0.88
2.19
2.47
2.78
1.96
0.95
1.00
0.86
2.90
2.82
0.97
3.06
1.11
3.44
1.80
1.23
3.15
1.21
10.0
9.5
9.0
8.5
8.0
7.5
7.0
6.5
6.0
5.5
5.0
4.5
4.0
3.5
3.0
2.5
2.0
1.5
1.0
0.5
0.0
f1 (ppm)

^{13}C 101 MHz

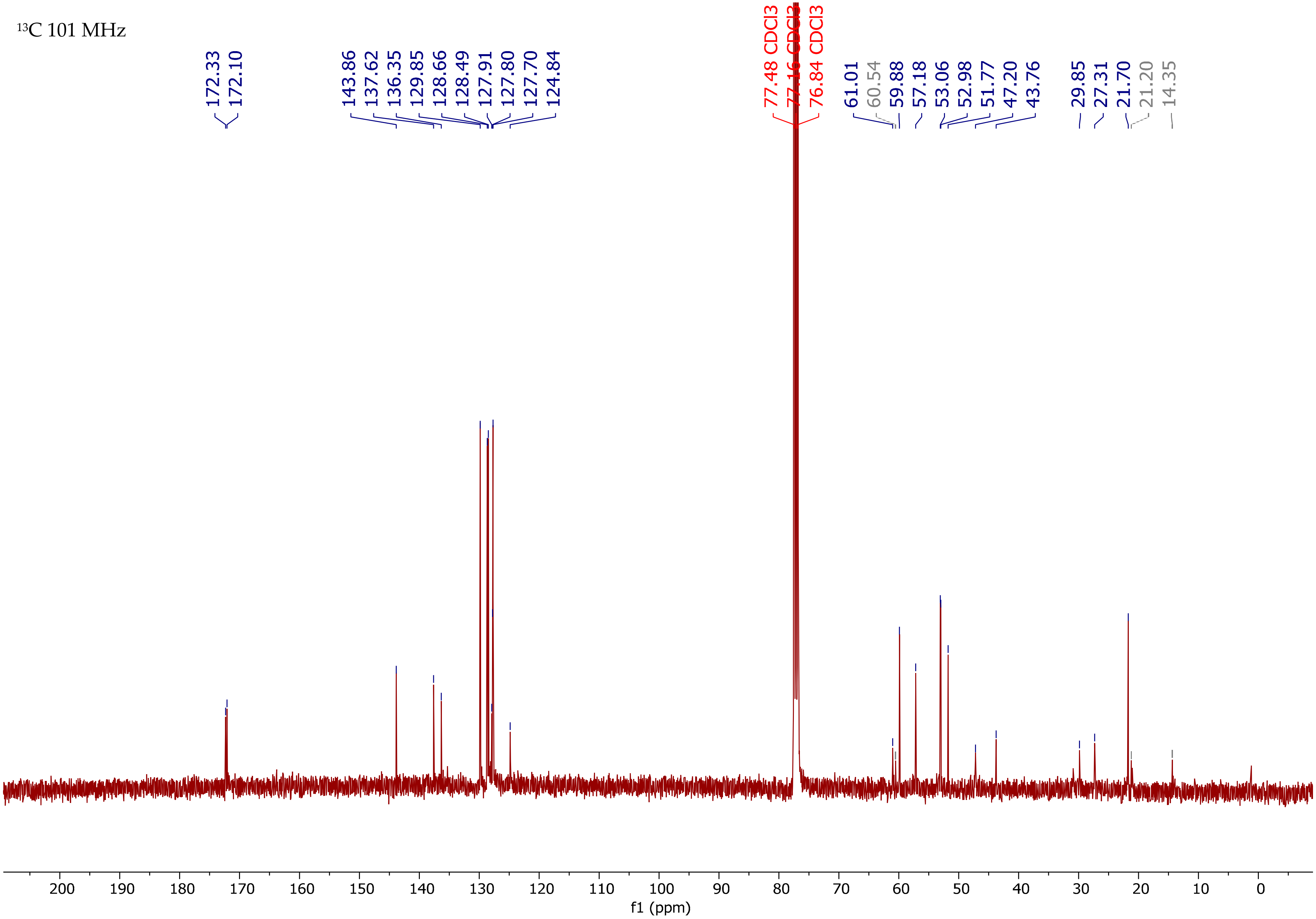

172.33
172.10
143.86
137.62
136.35
129.85
128.66
128.49
127.91
127.80
127.70
124.84
77.48 CDCl3
77.16 CDCl3
76.84 CDCl3
61.01
60.54
59.88
57.18
53.06
52.98
51.77
47.20
43.76
29.85
27.31
21.70
21.20
14.35
200 190 180 170 160 150 140 130 120 110 100 90 80 70 60 50 40 30 20 10 0
f1 (ppm)

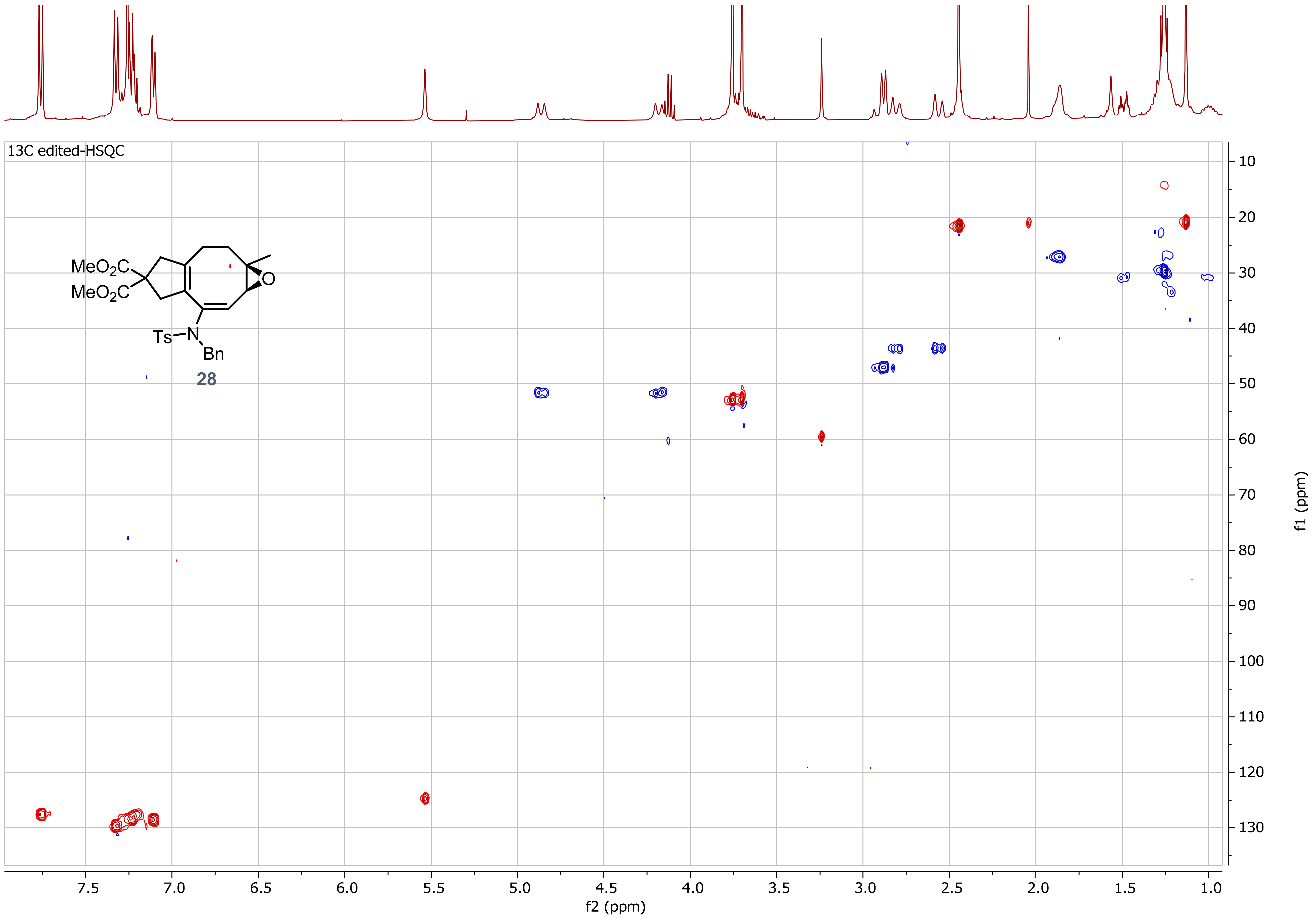
13C edited-HSQC
MeO2C
MeO2C
O
Ts
N
Bn
28
f2 (ppm)
f1 (ppm)

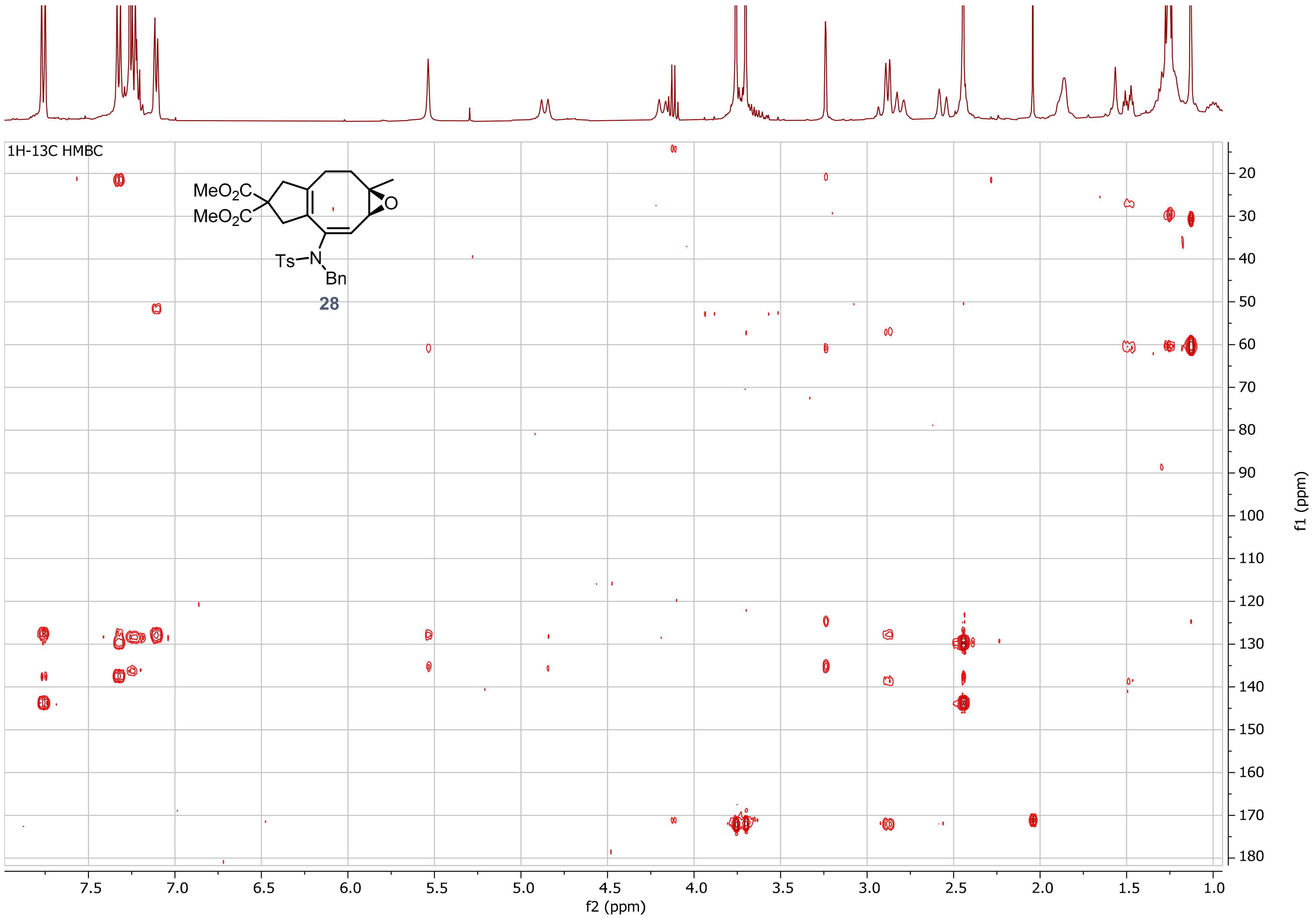
1H-13C HMBC
MeO2C
MeO2C
O
Ts
N
Bn
28
f2 (ppm)
f1 (ppm)

^{1}H 400 MHz

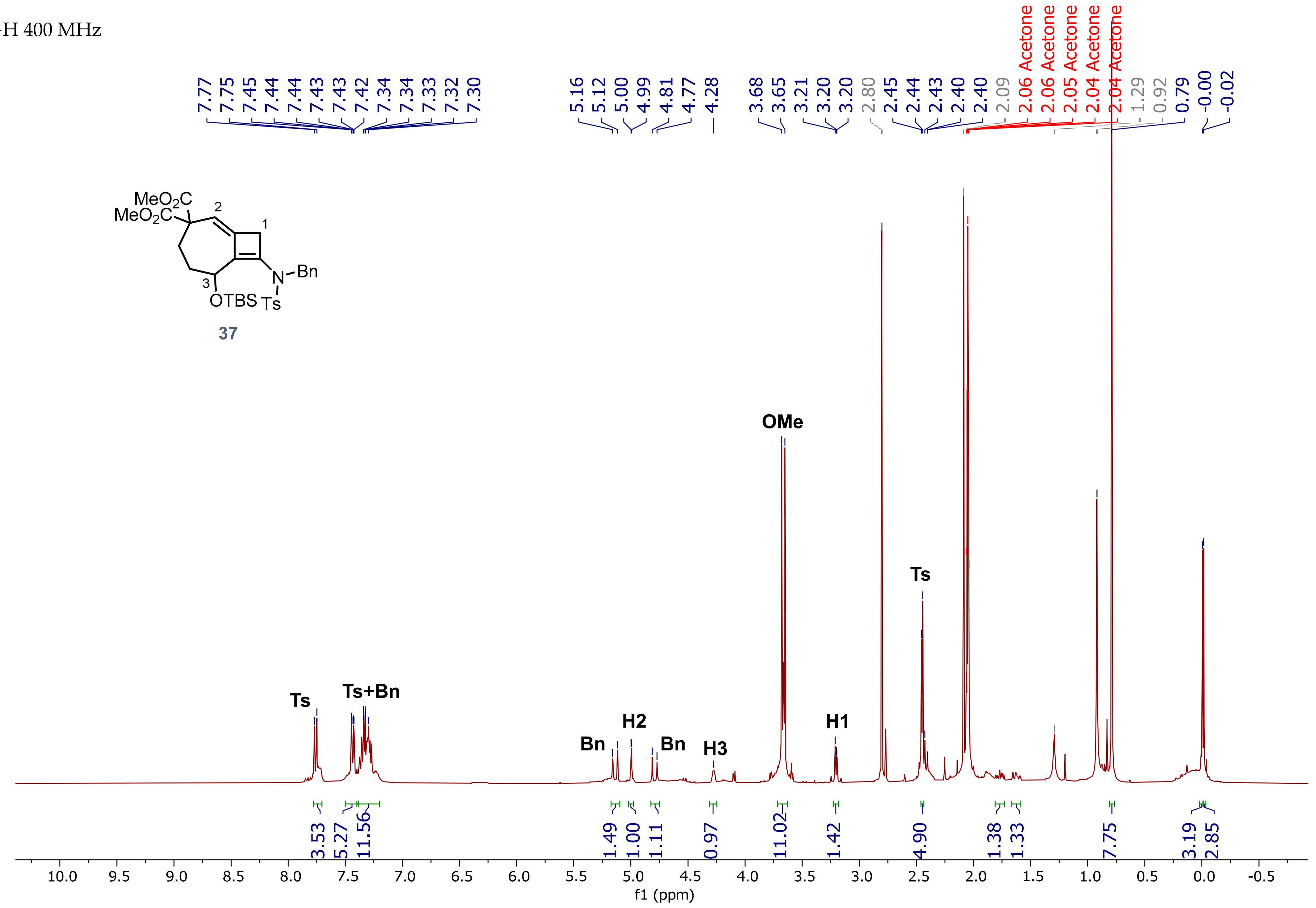

7.77 7.75 7.45 7.44 7.44 7.43 7.43 7.42 7.34 7.34 7.33 7.32 7.30
5.16 5.12 5.00 4.99 4.81 4.77 4.28
3.68 3.65 3.21 3.20 3.20 2.80 2.45 2.44 2.43 2.40 2.40 2.09
2.06 Acetone
2.06 Acetone
2.05 Acetone
2.04 Acetone
2.04 Acetone
1.29 0.92 0.79 -0.00 -0.02
MeO2C
MeO2C
2
1
3
N
Bn
OTBS
Ts
37
OMe
Ts
Ts
Ts+Bn
Bn
H2
Bn
H3
H1
3.53 5.27 11.56 1.49 1.00 1.11 0.97 11.02 1.42 4.90 1.38 1.33 7.75 3.19 2.85
10.0 9.5 9.0 8.5 8.0 7.5 7.0 6.5 6.0 5.5 5.0 4.5 4.0 3.5 3.0 2.5 2.0 1.5 1.0 0.5 0.0 -0.5
f1 (ppm)

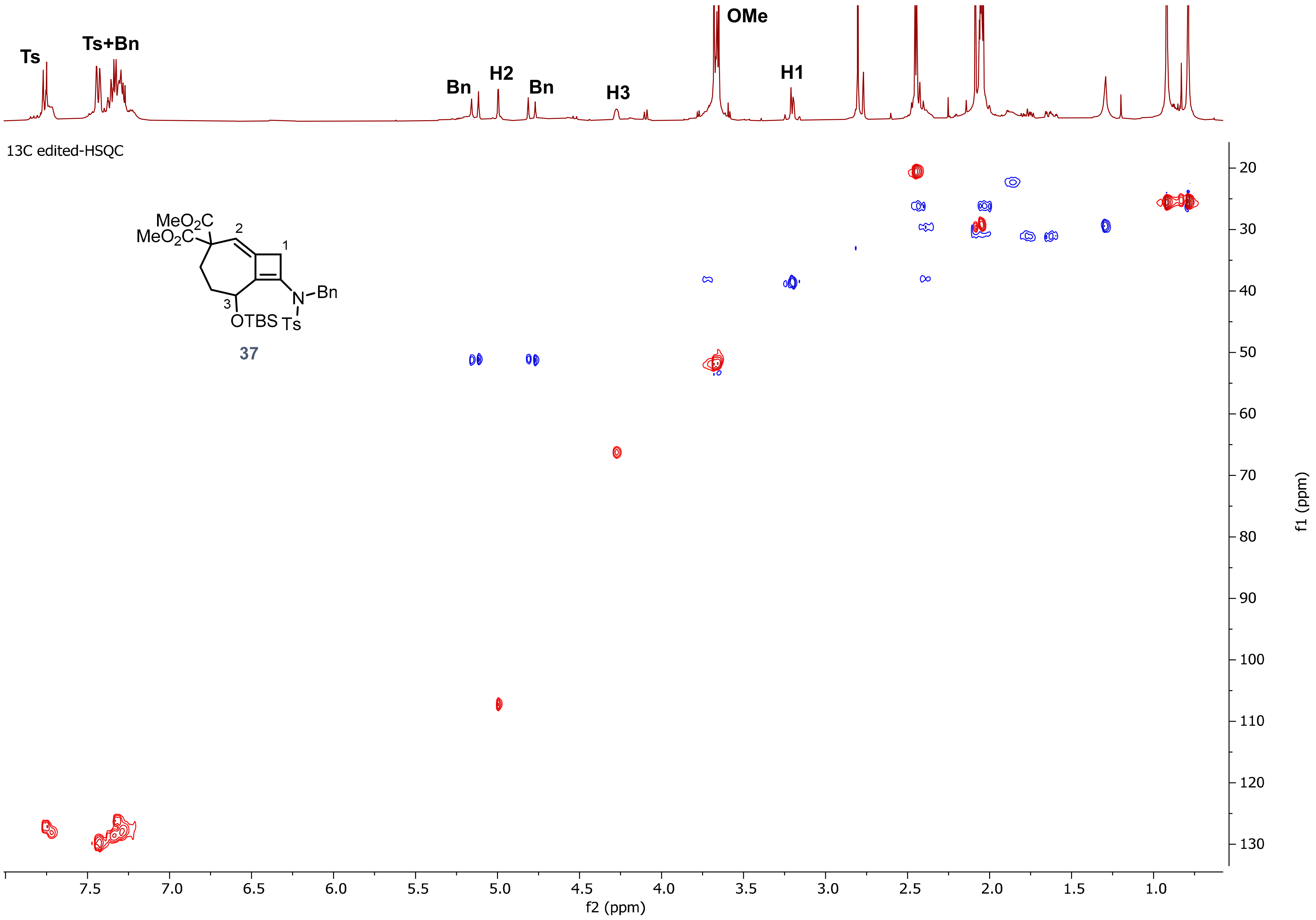
Ts
Ts+Bn
Bn
H2
Bn
H3
OMe
H1
13C edited-HSQC
MeO2C
MeO2C
2
1
N
Bn
3
OTBS
Ts
37
f2 (ppm)
f1 (ppm)

^{1}H 400 MHz

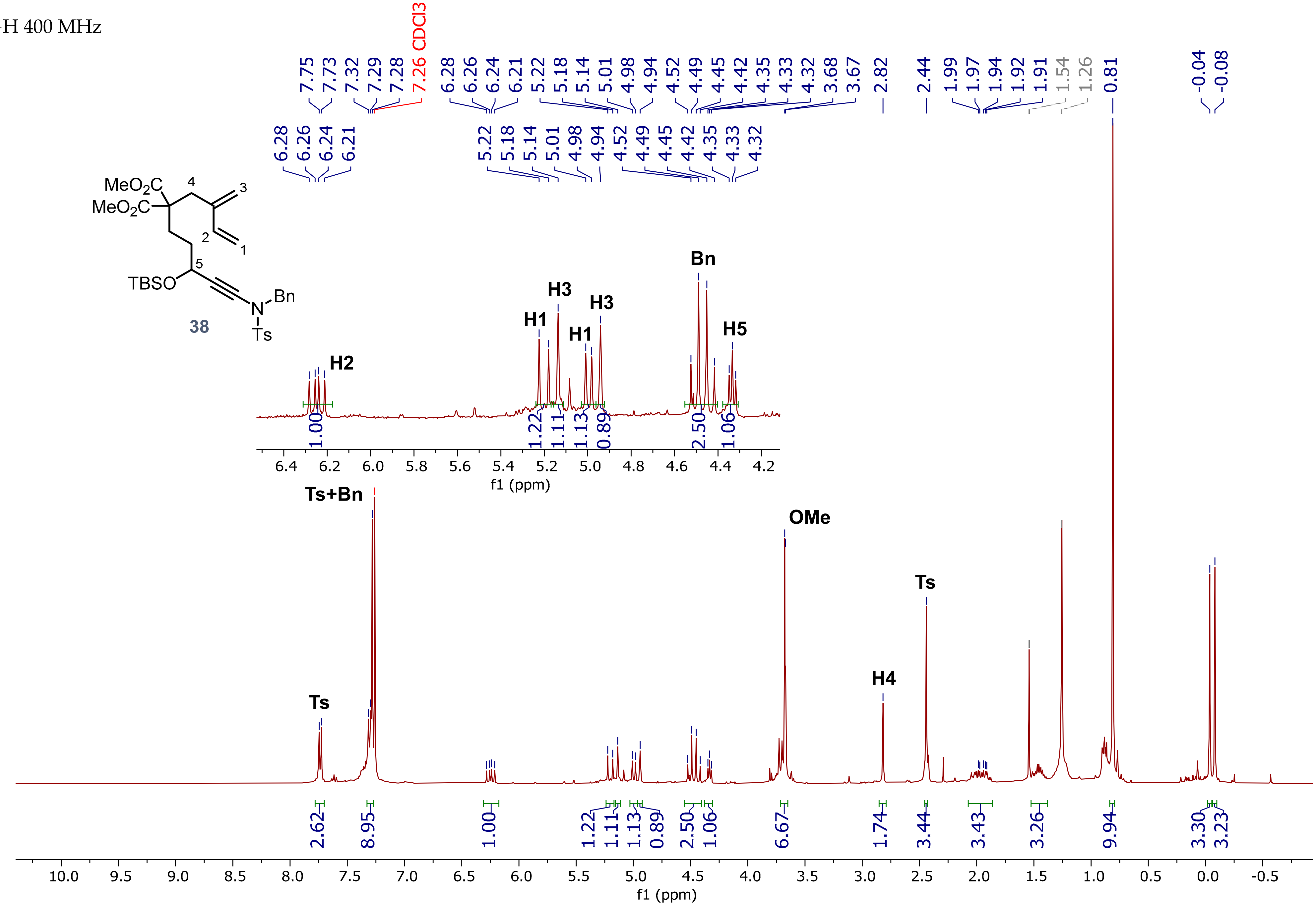

7.75
7.73
7.32
7.29
7.28
7.26 CDCl3
6.28
6.26
6.24
6.21
5.22
5.18
5.14
5.01
4.98
4.94
4.52
4.49
4.45
4.42
4.35
4.33
4.32
3.68
3.67
2.82
2.44
1.99
1.97
1.94
1.92
1.91
1.54
1.26
0.81
-0.04
-0.08
MeO2C
MeO2C
TBSO
N
Bn
Ts
1
2
3
4
5
38
H2
H1
H3
H1
H3
Bn
H5
1.00
1.22
1.11
1.13
0.89
2.50
1.06
Ts+Bn
OMe
Ts
H4
Ts
2.62
8.95
1.00
1.22
1.11
1.13
0.89
2.50
1.06
6.67
1.74
3.44
3.43
3.26
9.94
3.30
3.23
f1 (ppm)

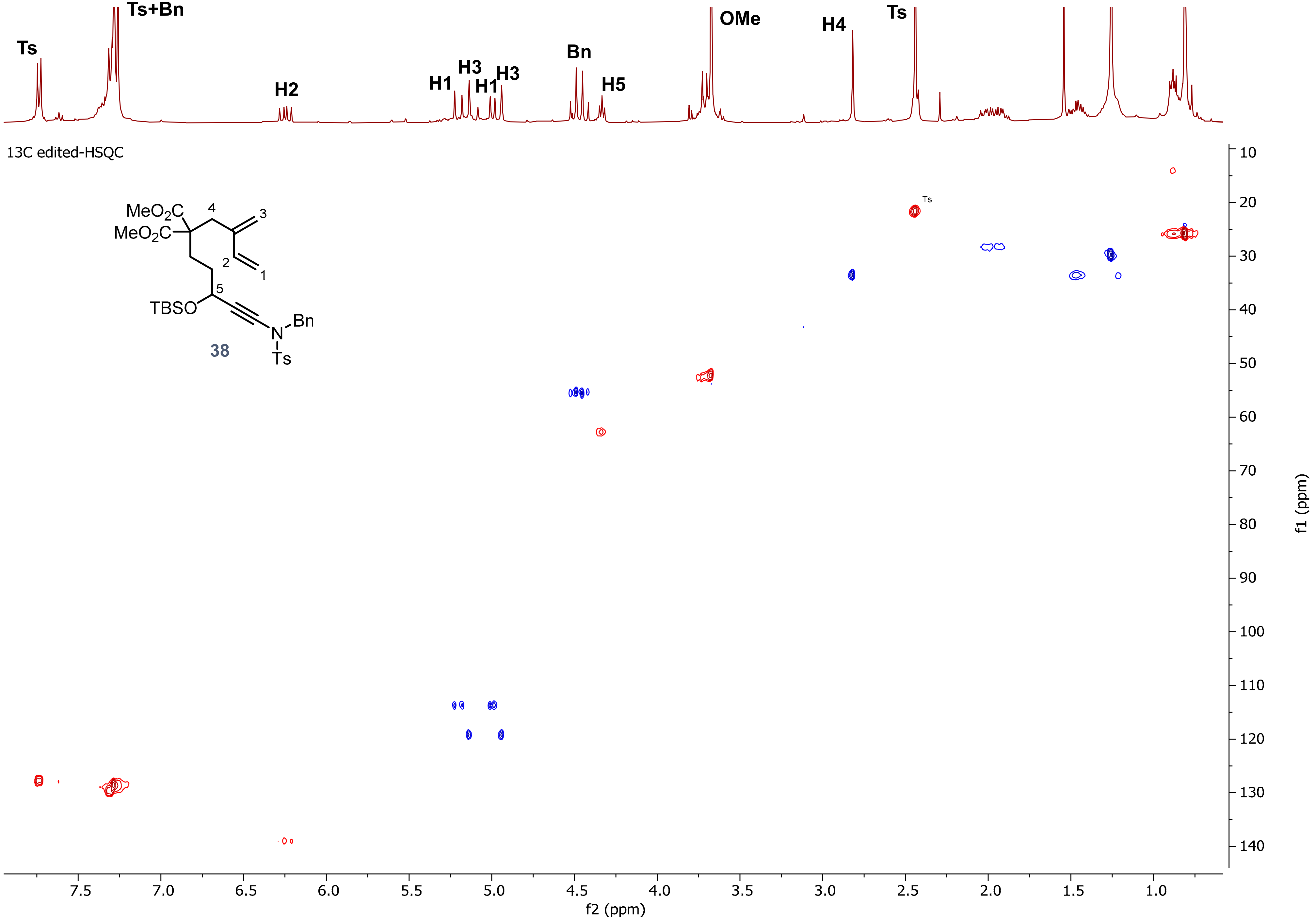

Ts
Ts+Bn
H2
H1
H3
H1
H3
Bn
H5
OMe
H4
Ts
13C edited-HSQC
MeO2C
MeO2C
4
3
2
1
5
TBSO
N
Bn
Ts
38
f2 (ppm)
f1 (ppm)

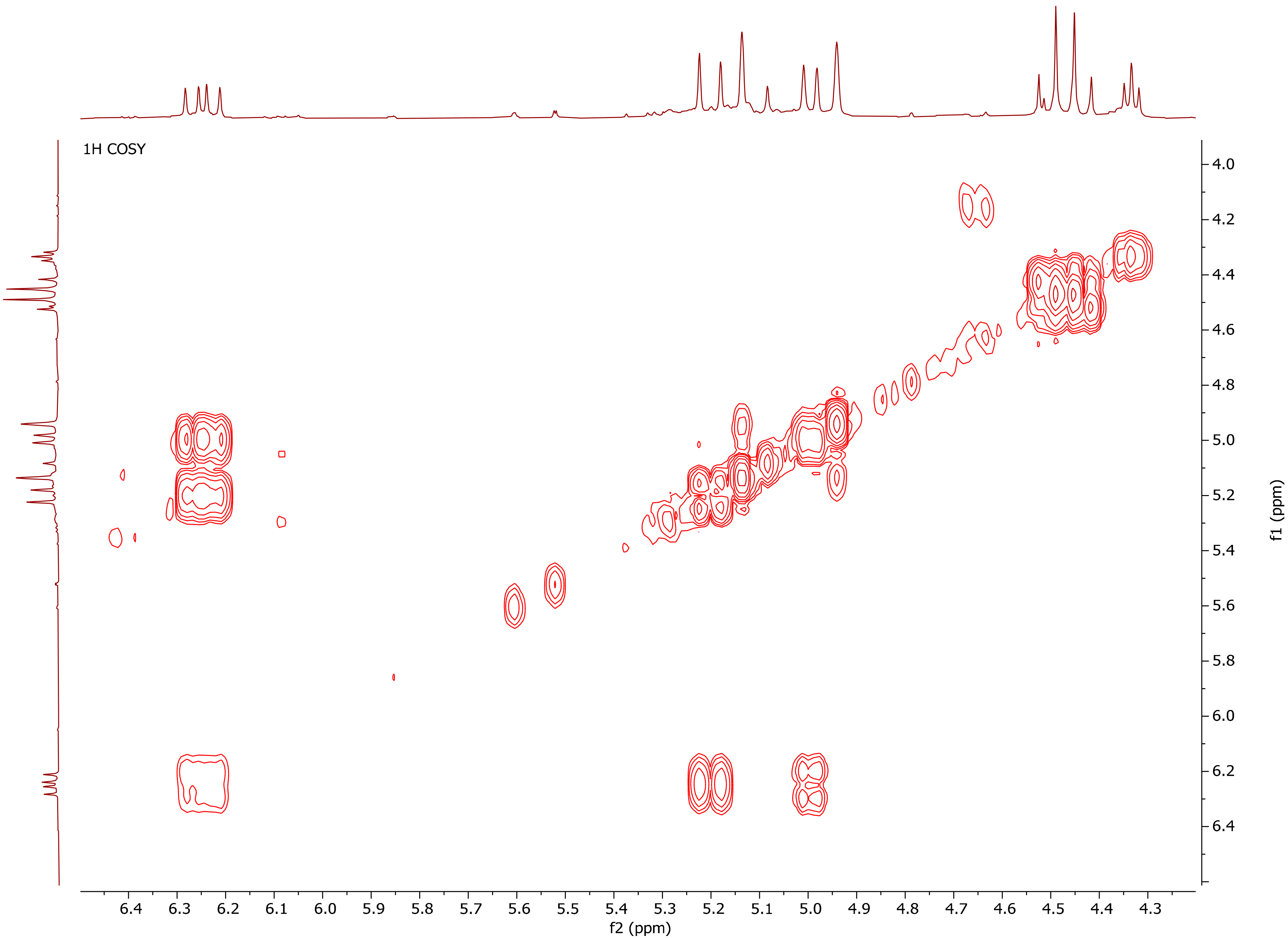
1H COSY
f2 (ppm)
f1 (ppm)
6.4
6.3
6.2
6.1
6.0
5.9
5.8
5.7
5.6
5.5
5.4
5.3
5.2
5.1
5.0
4.9
4.8
4.7
4.6
4.5
4.4
4.3
4.0
4.2
4.4
4.6
4.8
5.0
5.2
5.4
5.6
5.8
6.0
6.2
6.4

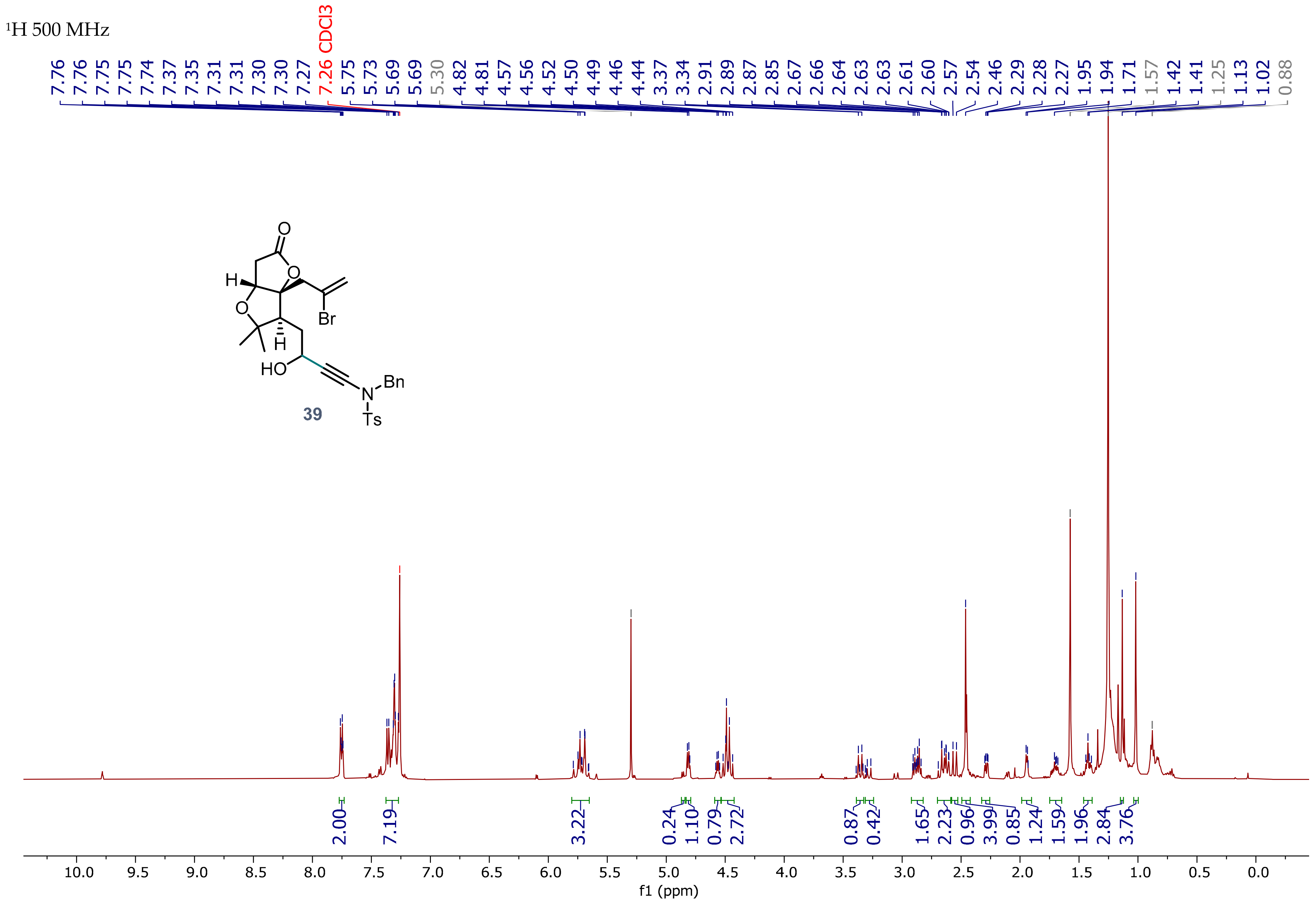

^{1}H 500 MHz
7.76 7.76 7.75 7.75 7.74 7.37 7.35 7.31 7.31 7.30 7.30 7.27 7.26 CDCl3 5.75 5.73 5.69 5.69 5.30 4.82 4.81 4.57 4.56 4.52 4.50 4.49 4.46 4.44 3.37 3.34 2.91 2.89 2.87 2.85 2.67 2.66 2.64 2.63 2.63 2.61 2.60 2.57 2.54 2.46 2.29 2.28 2.27 1.95 1.94 1.71 1.57 1.42 1.41 1.25 1.13 1.02 0.88
O
H
O
Br
O
H
HO
Bn
N
Ts
39
2.00 7.19 3.22 0.24 1.10 0.79 2.72 0.87 0.42 1.65 2.23 0.96 3.99 0.85 1.24 1.59 1.96 2.84 3.76
10.0 9.5 9.0 8.5 8.0 7.5 7.0 6.5 6.0 5.5 5.0 4.5 4.0 3.5 3.0 2.5 2.0 1.5 1.0 0.5 0.0
f1 (ppm)

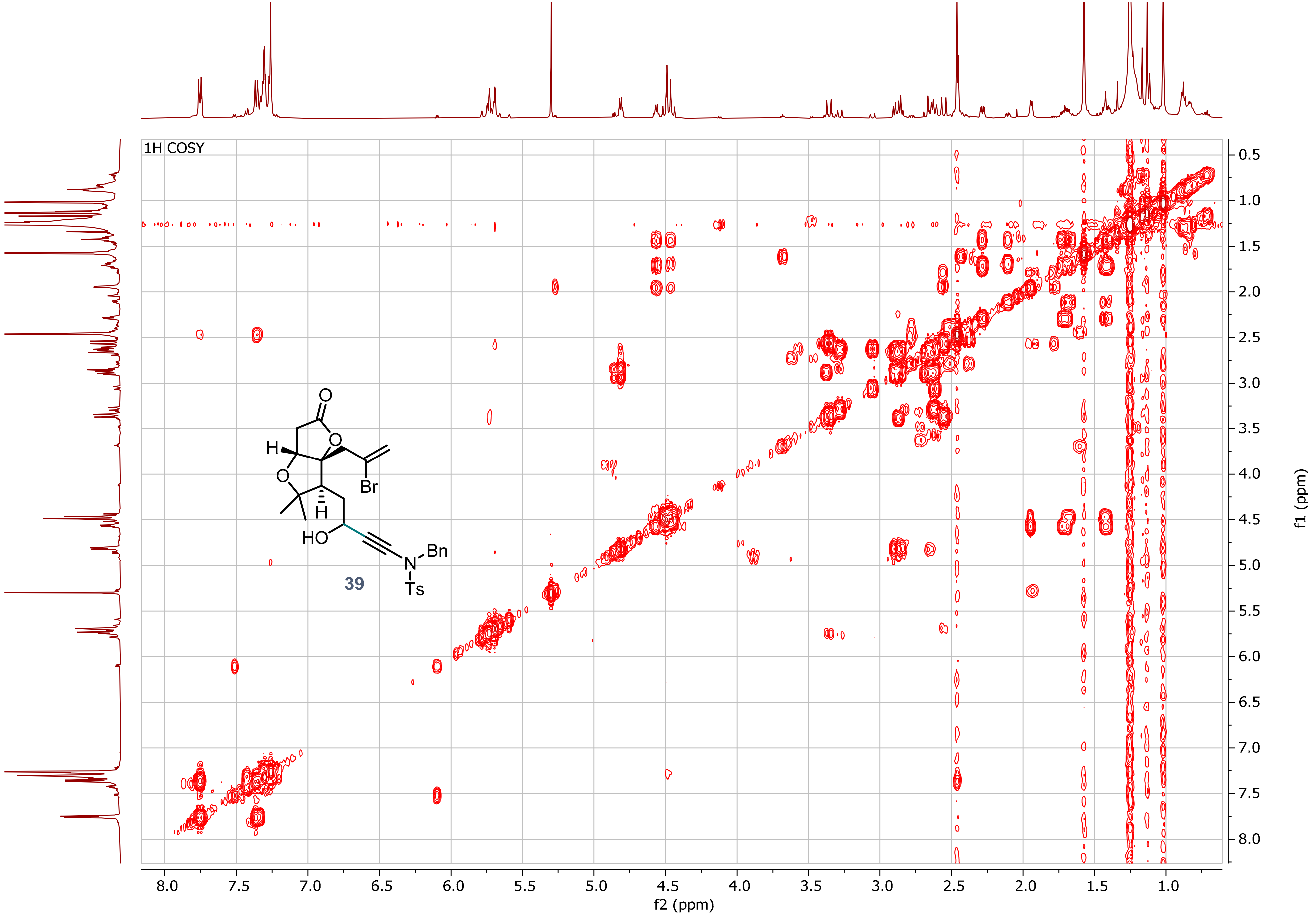
1H COSY
O
H
O
O
Br
H
HO
N
Bn
Ts
39
8.0
7.5
7.0
6.5
6.0
5.5
5.0
4.5
4.0
3.5
3.0
2.5
2.0
1.5
1.0
f2 (ppm)
0.5
1.0
1.5
2.0
2.5
3.0
3.5
4.0
4.5
5.0
5.5
6.0
6.5
7.0
7.5
8.0
f1 (ppm)

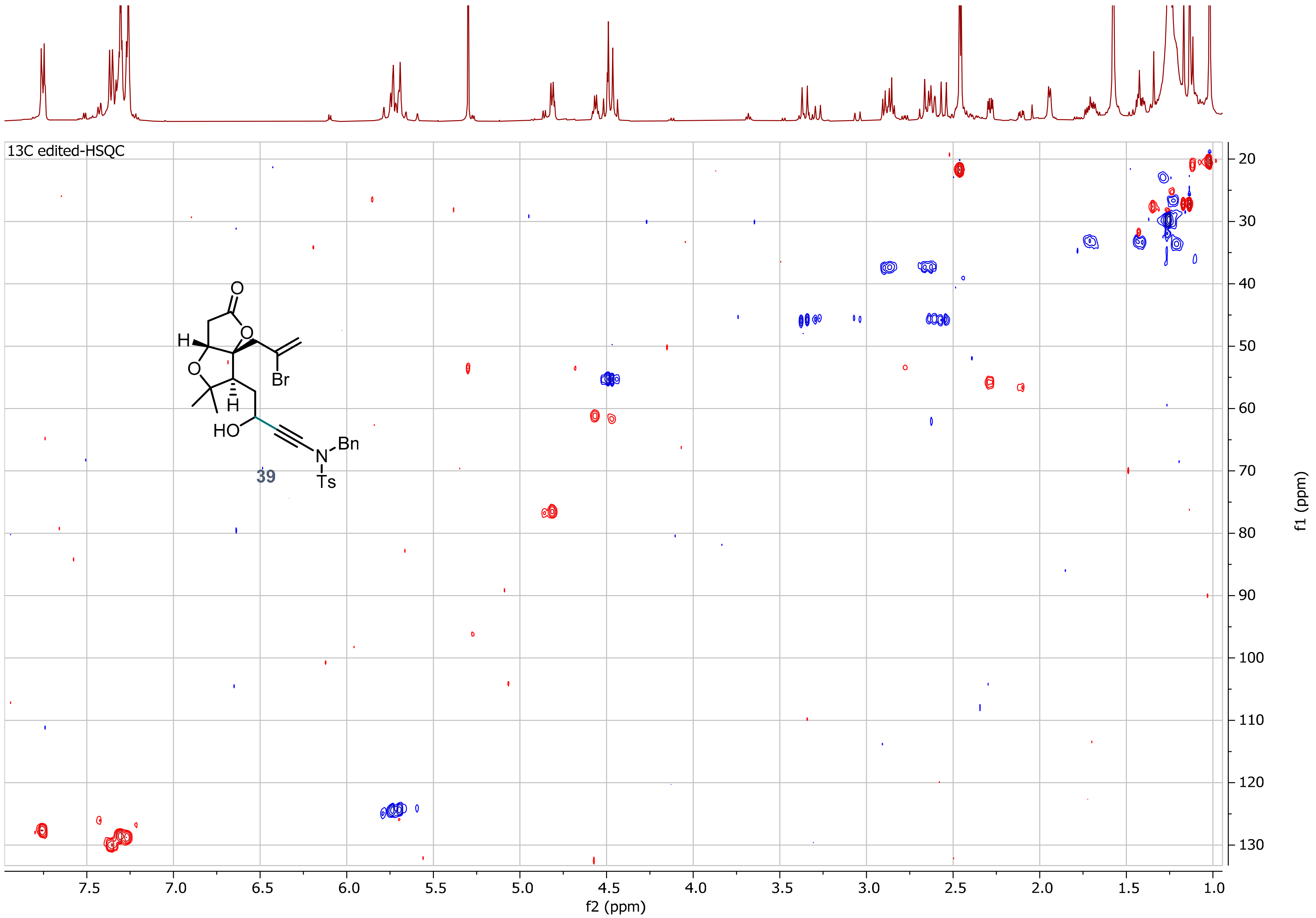
13C edited-HSQC
H
O
O
O
Br
H
HO
Bn
N
Ts
39
f2 (ppm)
f1 (ppm)
